# Distal Bias of Meiotic Crossovers in Hexaploid Bread Wheat Reflects Spatio-Temporal Asymmetry of the Meiotic Program

**DOI:** 10.3389/fpls.2021.631323

**Published:** 2021-02-12

**Authors:** Kim Osman, Uthman Algopishi, James D. Higgins, Ian R. Henderson, Keith J. Edwards, F. Chris H. Franklin, Eugenio Sanchez-Moran

**Affiliations:** ^1^School of Biosciences, University of Birmingham, Birmingham, United Kingdom; ^2^Department of Genetics and Genome Biology, University of Leicester, Leicester, United Kingdom; ^3^Department of Plant Sciences, University of Cambridge, Cambridge, United Kingdom; ^4^School of Biological Sciences, University of Bristol, Bristol, United Kingdom

**Keywords:** *Triticum aestivum* (bread wheat), meiosis, recombination, crossovers, distal bias, DNA double-strand breaks, immunolocalization

## Abstract

Meiotic recombination generates genetic variation and provides physical links between homologous chromosomes (crossovers) essential for accurate segregation. In cereals the distribution of crossovers, cytologically evident as chiasmata, is biased toward the distal regions of chromosomes. This creates a bottleneck for plant breeders in the development of varieties with improved agronomic traits, as genes situated in the interstitial and centromere proximal regions of chromosomes rarely recombine. Recent advances in wheat genomics and genome engineering combined with well-developed wheat cytogenetics offer new opportunities to manipulate recombination and unlock genetic variation. As a basis for these investigations we have carried out a detailed analysis of meiotic progression in hexaploid wheat (*Triticum aestivum*) using immunolocalization of chromosome axis, synaptonemal complex and recombination proteins. 5-Bromo-2′-deoxyuridine (BrdU) labeling was used to determine the chronology of key events in relation to DNA replication. Axis morphogenesis, synapsis and recombination initiation were found to be spatio-temporally coordinated, beginning in the gene-dense distal chromosomal regions and later occurring in the interstitial/proximal regions. Moreover, meiotic progression in the distal regions was coordinated with the conserved chromatin cycles that are a feature of meiosis. This mirroring of the chiasma bias was also evident in the distribution of the gene-associated histone marks, H3K4me3 and H3K27me3; the repeat-associated mark, H3K27me1; and H3K9me3. We believe that this study provides a cytogenetic framework for functional studies and ongoing initiatives to manipulate recombination in the wheat genome.

## Introduction

Utilizing the genetic variation that arises from meiotic recombination plays a pivotal role in crop improvement programs. Although substantial progress has been made in recent decades in the improvement of yield of major crops such as wheat, rice, and maize, the existential threat of climate change introduces additional demands for crops that are sufficiently robust to maintain yield in the face of biotic and abiotic challenges (Halford et al., 2015). This is exacerbated by an increase in human population and individual wealth within many countries which places additional demands on food production (Hickey et al., 2019). Hexaploid wheat is the most widely grown cereal crop, currently accounting for 20% of the calories and protein consumed by humans and an important source of vitamins and micronutrients (Shewry, 2009). The recent establishment of a fully-annotated bread wheat reference genome and ensuing genomics resources promises to revolutionize functional studies and trait discovery for the improvement of crop varieties (IWGSC, 2018; Adamski et al., 2020). In order to fully benefit from the new technological developments and face future challenges a thorough understanding of the wheat meiotic recombination pathway will also be required.

Gamete formation in most sexually reproducing organisms is achieved through meiosis, a specialized form of cell-division in which S-phase is followed by two sequential rounds of nuclear division. During prophase I of meiosis homologous recombination (HR) between maternal and paternal chromosomes results in the reciprocal exchange of genetic information to form genetic crossovers (COs), which are manifested cytologically as chiasmata. CO formation gives rise to novel allelic combinations thereby generating genetic variation and is essential for accurate segregation of the homologous chromosomes (homologs) at the first meiotic division. A subsequent second division separates the sister chromatids to form haploid gametes.

Meiotic HR is initiated by the programmed formation of numerous DNA double-strand breaks (DSBs) catalyzed by the SPO11 complex supported by various accessory proteins (Lam and Keeney, 2014). In Arabidopsis, the SPO11 complex comprises two A subunits (SPO11-1 and SPO11-2) and two B subunits (MTOPVIB), forming a heteromeric complex (Stacey et al., 2006; Vrielynck et al., 2016). The genomic distribution of DSBs is non-random, preferentially forming in short regions referred to as DSB hotspots (Baudat and Nicolas, 1997; Smagulova et al., 2011; Choi et al., 2013). In Arabidopsis and maize meiotic DSB hotspots are associated with open chromatin, occurring in regions of low nucleosome density in gene promoters and specific classes of transposons, but differ from mammalian hotspots in their complex relationship with the open chromatin mark histone H3 lysine 4 tri-methyl (H3K4me3) (Choi et al., 2018). DSBs are resected by the MRX/N complex to reveal single-stranded DNA overhangs that are bound by RPA, followed by the strand invasion proteins RAD51 and DMC1 (Osman et al., 2011). To ensure that a proportion of the DSBs are repaired as CO products, the initial RAD51/DMC1 catalyzed strand-exchange stage is biased toward use of the homologous chromosome as the repair template (Schwacha and Kleckner, 1997). In plants, fewer than 10% of the DSBs are repaired as COs and the remainder as non-COs (Mercier et al., 2015). Repair is controlled such that a minimum of one, obligate, CO per homolog pair (bivalent) is formed (Jones and Franklin, 2006). Additional COs are subject to a patterning phenomenon known as CO interference, which results in COs being well-spaced along chromosomes (Jones and Franklin, 2006). In Arabidopsis, formation of these Class I COs, which amount to around 85% of total COs, requires the activities of the ZMM recombination proteins: Zip2/SHOC1, Zip3/HEI10, ZIP4, MSH4, MSH5, and MER3 (Higgins et al., 2004, 2008b; Mercier et al., 2005; Chelysheva et al., 2007, 2012; Macaisne et al., 2008, 2011). The remaining COs (Class II) are not sensitive to interference and in part, require the activity of MUS81 recombinase (Higgins et al., 2008a).

HR is accompanied by programmed remodeling of the meiotic chromosomes (Zickler and Kleckner, 1999). Following S-phase, pairs of sister chromatids are linked by cohesin proteins (Haering and Jessberger, 2012). At the onset of leptotene, the sister chromatids become organized into linear looped arrays that are conjoined at the loop bases by a proteinaceous axis that is elaborated along their length (Zickler and Kleckner, 1999). As leptotene transitions into zygotene, the homologs progressively align before coming into close apposition through the formation of the synaptonemal complex (SC) (Zickler and Kleckner, 1998). The SC has a tripartite structure comprising the chromosome axes, now referred to as lateral elements, cross-linked by overlapping transverse filament proteins (Page and Hawley, 2004). At pachytene the SC is fully polymerized along the length of the synapsed homologs. By diplotene CO formation is completed, the SC disassembles and the homologs become progressively condensed appearing at diakinesis as bivalents linked by one or more chiasmata. At metaphase I the bivalents align on the equator prior to the first meiotic division. Mutant analysis in a wide variety of organisms including plants has found that HR and meiotic chromosome remodeling are highly interdependent (Osman et al., 2011; Mercier et al., 2015).

One of the limitations in most crop species is that meiotic CO frequency is rather low, typically 1–3 COs per bivalent (Higgins et al., 2014). In addition, in many species CO distribution exhibits a tendency to localize in particular chromosomes regions, often favoring distal regions (Jones, 1984). This is particularly evident in cereals with large genomes, for example wheat and barley, where a strong distal CO bias limits their formation in interstitial and proximal chromosome regions amounting to 50–70% of the overall genome (Choulet et al., 2014; Higgins et al., 2014).

Studies in Arabidopsis have revealed that it is possible to significantly enhance the level of Class II COs through the mutation of anti-recombination genes, *FANCM, RECQ4*, and *FIGL1* (Crismani et al., 2012; Girard et al., 2015; Séguéla-Arnaud et al., 2015). In other work in Arabidopsis the meiotic E3 ligase, *HEI10*, has been found to regulate the level of Class I interfering COs (Chelysheva et al., 2012; Ziolkowski et al., 2017; Serra et al., 2018). When *HEI10* over-expression was combined with *recq4a* and *recq4b* mutations the combined number of Class I and Class II COs was boosted from an average of 7.5–31 in individual F_2_ plants (Serra et al., 2018). Mutation of the recombination suppressor genes has been investigated in three crop species, rice (*Oryza sativa*), pea (*Pisum sativum*), and tomato (*Solanum lycopersicum*) where it was found that mutation of *recq4* increased COs by a factor of ~3-fold (Mieulet et al., 2018). Whether a similar impact on CO frequency will occur in large genome crops such as wheat and barley remains to be determined. Also, the hyper-rec mutants exhibit some evidence of reduced fertility and meiotic defects, which may prove more problematic in species with larger genomes (Fernandes et al., 2018). Furthermore, it appears that recombination-cold proximal/pericentromeric regions of chromosomes are relatively insensitive to the effects of hyper-rec mutants and *HEI10* overexpression (Serra et al., 2018).

Thus, despite substantial progress in manipulating meiotic CO frequency, effective application of these and other approaches such as targeting DSB sites will need refining if they are to be successfully employed in species such as wheat and barley, underlining the requirement for a detailed understanding of the meiotic pathway in these species. In a previous study we investigated meiotic progression in barley (Higgins et al., 2012). Unlike barley which is a diploid species, bread wheat, *Triticum aestivum*, is an allohexaploid, with 3 sub-genomes A, B, and D resulting from a double polyploidization process involving three related species (Sears, 1948). Despite being hexaploid, the presence of the *Ph1* locus enables bread wheat to behave as a diploid during meiosis by its influence on pairing of the homoeologous chromosomes and recombination (Riley and Chapman, 1958; Sears and Okamoto, 1958). The role of the *Ph1* locus has been studied extensively and was suggested to be two-fold. First, it was proposed that a cluster of Cdk2-like and S-adenosyl methionine-dependent methyltransferase (SAM-MTase) genes within the locus promote homologous chromosome pairing through an effect on chromatin structure and histone H1 phosphorylation and an associated change in the rate of pre-meiotic replication and subsequent synapsis (Greer et al., 2012; Rey and Prieto, 2014; Martín et al., 2017). Second, a paralog of the ZMM gene *ZIP4* within the *Ph1* region was reported to promote the maturation of late recombination complexes to form homologous COs (Martín et al., 2014, 2017; Rey et al., 2017). It now appears that the *ZIP4* paralogue may be responsible for most, if not all, of the *Ph1* effect (Rey et al., 2018). Apart from the analysis of *Ph1*, functional studies of wheat meiotic genes remain limited, with little over 10% of those described in other plant species (notably Arabidopsis, rice and maize), having been analyzed even to a limited degree (Da Ines et al., 2020). Nevertheless, the availability of TILLING populations and gene editing techniques is enabling progress as evidenced by recent analysis of *T. aestivum SPO11-2* and *T. turgidum MSH4/5* (Benyahya et al., 2020; Desjardins et al., 2020).

Here we present a detailed cytological overview of the meiotic program in Cadenza, a widely-used research variety with an EMS-induced TILLING mutant population (Rakszegi et al., 2010; Krasileva et al., 2017). We investigate how chromosome remodeling throughout prophase I is integrated with the recombination machinery and show that there is a spatio-temporal bias in the initiation and progression of recombination that mirrors the tendency of chiasmata/COs to occur in gene-dense distal regions of the chromosomes. We establish a time-frame for the duration of meiosis and confirm that wheat chromatin undergoes cycles of contraction and expansion during prophase I, as previously observed in barley and other species. Finally, we note and discuss interesting features of ASY1 and ZYP1 protein localization during the meiotic program. We believe this study will provide a reference framework for CO modification initiatives and functional studies of meiotic recombination for the benefit of crop improvement.

## Materials and Methods

### Plant Material

*T. aestivum* cv. Cadenza was obtained from www.SeedStor.ac.uk. Plants were grown in a controlled environment with photoperiod 16 h, temperature 20°C and relative humidity 60%.

### Antibody Production

AtHEI10 amino-acid residues 1–183 was expressed as a recombinant protein and purified from *E. coli*. Antibody was raised in rabbit (PTU/BS, Scottish National Blood Transfusion Service, now www.orygen.co.uk). Anti-TaCENH3 was raised in rabbit against a 19-residue peptide from the N-terminal of the protein [ARTKHPAVRKTKAPPKKQL-[C]-amide] conjugated to KLH (www.crbdiscovery.com).

### Cytological Procedures

Meiotic chromosome spreads were prepared from anthers isolated at the required stage of meiosis. For chiasma counts anthers were fixed and slides prepared according to Howell and Armstrong (2013) with minor modifications: anthers were macerated in 70% acetic acid and incubated for 1 min on a 45°C hot-plate before fixing with 130 μl cold fixative (3 parts of absolute ethanol: 1 part of glacial acetic acid) and staining with 5 μg ml^−1^ 4′,6-diamidino-2-phenylindole (DAPI) in Vectashield (Vector Labs). For immunolocalization, slides were prepared as described for *Brassica oleracea* in Armstrong et al. (2002) with the following modifications: ~20 anthers were digested in 20 μl enzyme mix (0.4% cytohelicase, 1.5% sucrose, 1% polyvinylpyrrolidone) in a cavity slide inside a humidified chamber at 37°C. After 4 min anthers were gently crushed to release pollen mother cells (PMCs), anther debris was quickly removed with a needle and digestion continued for a further 3 min. Up to 4 slides were prepared from each 20 μl digestion mix and PMCs were accurately staged using anti-AtASY1 and anti-AtZYP1 antibodies. Primary antibodies were used at the following dilutions: anti-AtASY1 rat, rabbit or guinea-pig, 1:500 (Armstrong et al., 2002); anti-AtZYP1 rabbit or guinea-pig, 1:500 (Higgins et al., 2005; Osman et al., 2018); anti-HsγH2A.X rabbit, 1:100 (Millipore); anti-AtDMC1 rabbit, 1:200 (Sanchez-Moran et al., 2007); anti-AtRAD51 rabbit, 1:200 (Mercier et al., 2003); anti-AtMSH4 rabbit, 1:200 (Higgins et al., 2004); anti-AtMSH5 rabbit, 1:200 (Higgins et al., 2008b); anti-AtHEI10 rabbit and HvHEI10 rabbit, 1:200 (see above and Lambing et al., 2015; Desjardins et al., 2020); anti-HvMLH3 rabbit, 1:200 (Phillips et al., 2013); anti-TaCENH3 rabbit, 1:200 (see above); H3K4me3, H3K27me1, H3K27me3, and H3K9me3 rabbit, according to the manufacturer's guidelines (Diagenode). For combined immunofluorescence and fluorescence *in situ* hybridization (FISH) of telomeric repeat sequences, slides were first prepared as for immunolocalization and the primary antibody applied (see above). After incubation and washing to remove unbound serum, the primary antibody was blocked with a secondary antibody-biotin conjugate at 1:100 in 1% bovine serum albumin (BSA), in 1X phosphate buffered saline, 0.1% Triton X100 (PBST). Slides were incubated for 30 min at 37°C, washed 3 times with PBST and an Arabidopsis telomeric-repeat FISH probe labeled with digoxigenin (Armstrong et al., 2001) was applied as described in Armstrong (2013). Secondary antibodies were FITC (green), Alexa Fluor 350 (blue), Cy3 or Alexa Fluor 594 (red) conjugates (Sigma; Thermo Fisher). Nuclear size was determined according to Higgins et al. (2012) using NIS Elements software (Nikon).

### Meiotic Time Course

Up to 0.5 ml BrdU (10 mM in 1X PBS, 0.1% Tween 20) was injected into the cavity above a developing wheat spike (taken as time 0). The spike was subsequently harvested at a defined time point and anthers of an appropriate size for meiosis were excised and fixed in 3:1 ethanol:acetic acid. Eight time points spanning the entire meiotic program were analyzed, each using a different spike/plant, and two replicates were analyzed for each. For each time-point, all stages of the meiotic program were assessed for BrdU labeling. Slides were prepared as for chiasma counts (see above), then made ready for immunolocalization by a modification of Chelysheva et al. (2010): slides were heated in 10 mM citrate buffer pH 6 in a 850 W microwave for 45 s (taking care not to let the buffer boil), then immediately transferred to PBST for 10 min. Standard immunolocalization (see above) was then used to detect ASY1/ZYP1 and incorporated BrdU (Armstrong et al., 2002). BrdU labeling reagent, mouse anti-BrdU antibody and anti-mouse Ig-fluorescein were from Roche.

### Microscopy

Fluorescence microscopy was carried out using a Nikon Eclipse 90i microscope fitted with a Nikon DS-Qi1Mc camera. NIS Elements software (Nikon) was used to capture images as z-stacks with a 0.2 μM z-step and to carry out simple processing steps such as color balance adjustment and creation of composite images. For accuracy, chiasmata were interpreted and counted by examining all individual z-frames within a nucleus. Recombination foci were counted using single z-frames from the raw data files in order to clearly distinguish individual foci, confirm axis/SC-association and avoid the saturation of signal that can occur in composite images. All z-frames within a nucleus were counted - the count tool in NIS Elements was used to mark scored foci, thus preventing double counting when moving between frames. All in-focus foci (for the particular frame in question) were counted. Where necessary, the NIS Elements Gauss-Laplace sharpen tool was used to help resolve close-together foci. Any rare, aggregates of foci which could not be resolved at this level, were scored only once. An example image, with marked counted foci, is shown in Supplementary Figure 1.

### Statistics

Nuclear size and recombination foci count differences were tested for significance using single-factor Anova.

## Results

### Chiasmata Are Predominantly Distal in Hexaploid Wheat cv. Cadenza

**Figure 1:**
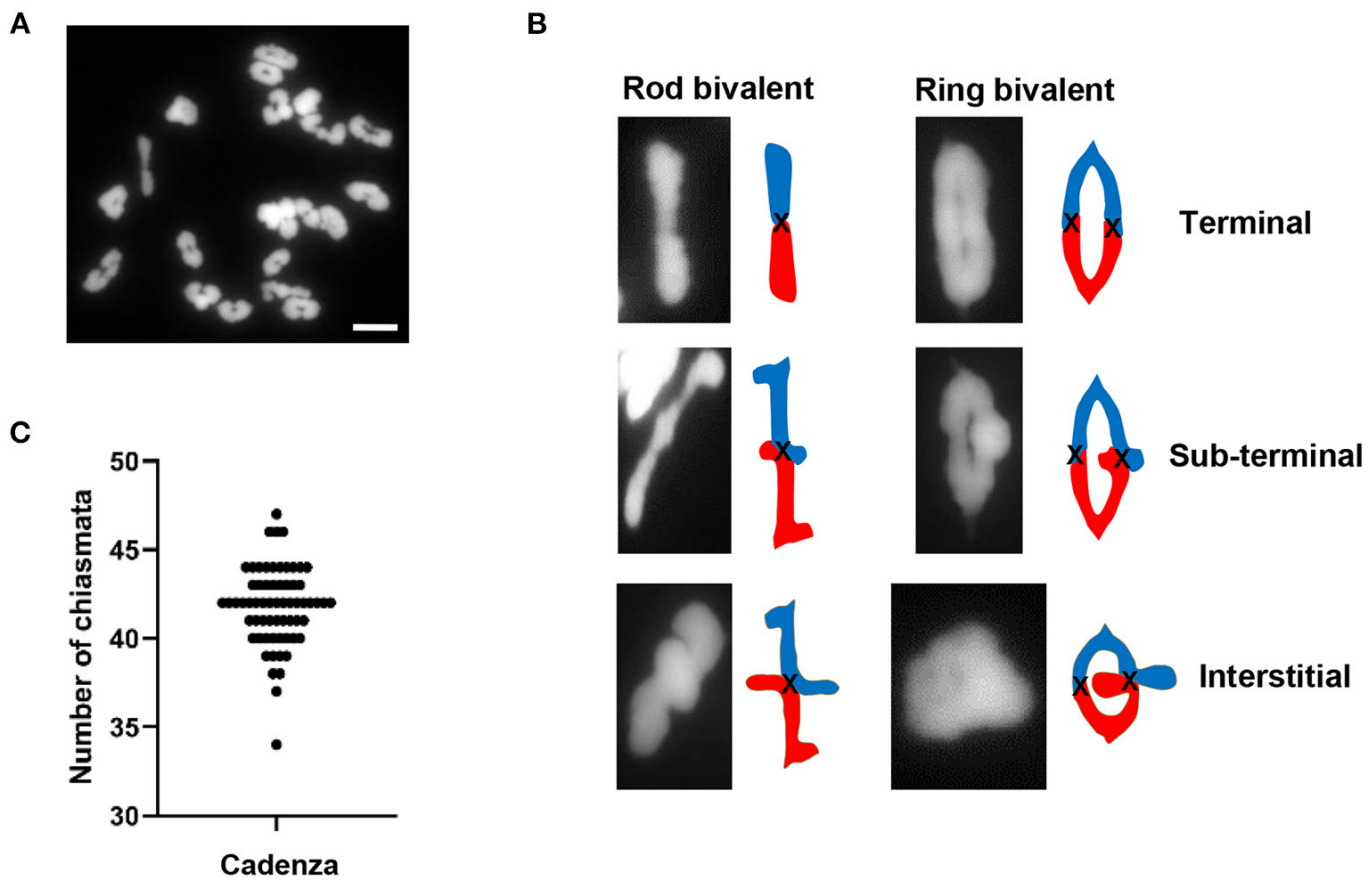
Chiasma frequency of *T. aestivum* cv. Cadenza. **(A)** Chromosome spread of metaphase I PMC showing 21 DAPI-stained bivalents. **(B)** Bivalent shapes at metaphase I indicate number and position of chiasmata. **(C)** Number of chiasmata per PMC (*n* = 64). Bar = 10 μM.

A cytological analysis of chiasma frequency and distribution was carried out in pollen mother cells (PMCs) of Cadenza, a UK spring wheat variety which forms the background for an EMS-induced TILLING mutant population (Rakszegi et al., 2010; Krasileva et al., 2017). Despite having three related sub-genomes (A, B, and D), hexaploid wheat (2*n* = 42) behaves as a diploid during meiosis due to the presence of the *Ph1* locus (Riley and Chapman, 1958; Sears and Okamoto, 1958), the major regulator of homoeologue pairing and recombination which ensures that recombination is restricted to true homologs rather than homoeologues (equivalent chromosomes from the other sub-genomes). Thus, Cadenza usually forms 21 bivalents at metaphase I (Figure 1A). Chiasmata, the cytological manifestation of COs, were interpreted according to bivalent shape at metaphase I, allowing a determination of their relative position along chromosomes (Sybenga, 1975). The vast majority of bivalents (93%, *n* = 1337) were “ring” bivalents, with at least one chiasma in each chromosome arm, while “rod” bivalents possessed chiasmata in only one arm (Figure 1B). The mean number of chiasmata per PMC was 41.8 ± 0.28 (*n* = 64) (Figure 1C) and the majority (88%) were distal (near the telomeres) (Figure 1B). Of these 76% were classified as terminal, as they could not be visually resolved from the telomeres in the highly condensed metaphase I bivalents. The remaining 12% were classified as sub-terminal, as they were close to, but clearly distinguishable from, the chromosome ends (Figure 1B).

### Chromosome Axis Formation and Synapsis Are Initially Polarized to the Distal Regions

During early prophase I, the telomeres of many species, including cereal grasses, cluster together and attach to the nuclear membrane in a highly conserved organization known as the “bouquet” (Chikashige et al., 1994). It was proposed that this configuration promotes initial contacts between homologous chromosomes, with subsequent alignment and synapsis facilitated by telomere-led movements driven by the cytoskeleton, although the functional significance of the bouquet is still a matter of debate (Zickler and Kleckner, 2016; Zeng et al., 2018).

Previous analysis of plant meiotic chromosomes using electron microscopy indicated that synapsis initiates in the distal chromosomal regions, close to the telomeres (Albini et al., 1984). More recently, immunolocalization using antibodies which recognize components of the meiotic chromosome axis and synaptonemal complex (SC) have enabled more detailed analysis of axis formation and synapsis in a range of plant species, including cereals (Golubovskaya et al., 2006; Mikhailova et al., 2006; Boden et al., 2009; Higgins et al., 2012; Khoo et al., 2012; Sepsi et al., 2017). For Cadenza, we combined immunofluorescence with fluorescence *in situ* hybridization (FISH) of telomeric repeat sequences to investigate axis morphogenesis and SC formation in conjunction with telomere dynamics.

**Figure 2:**
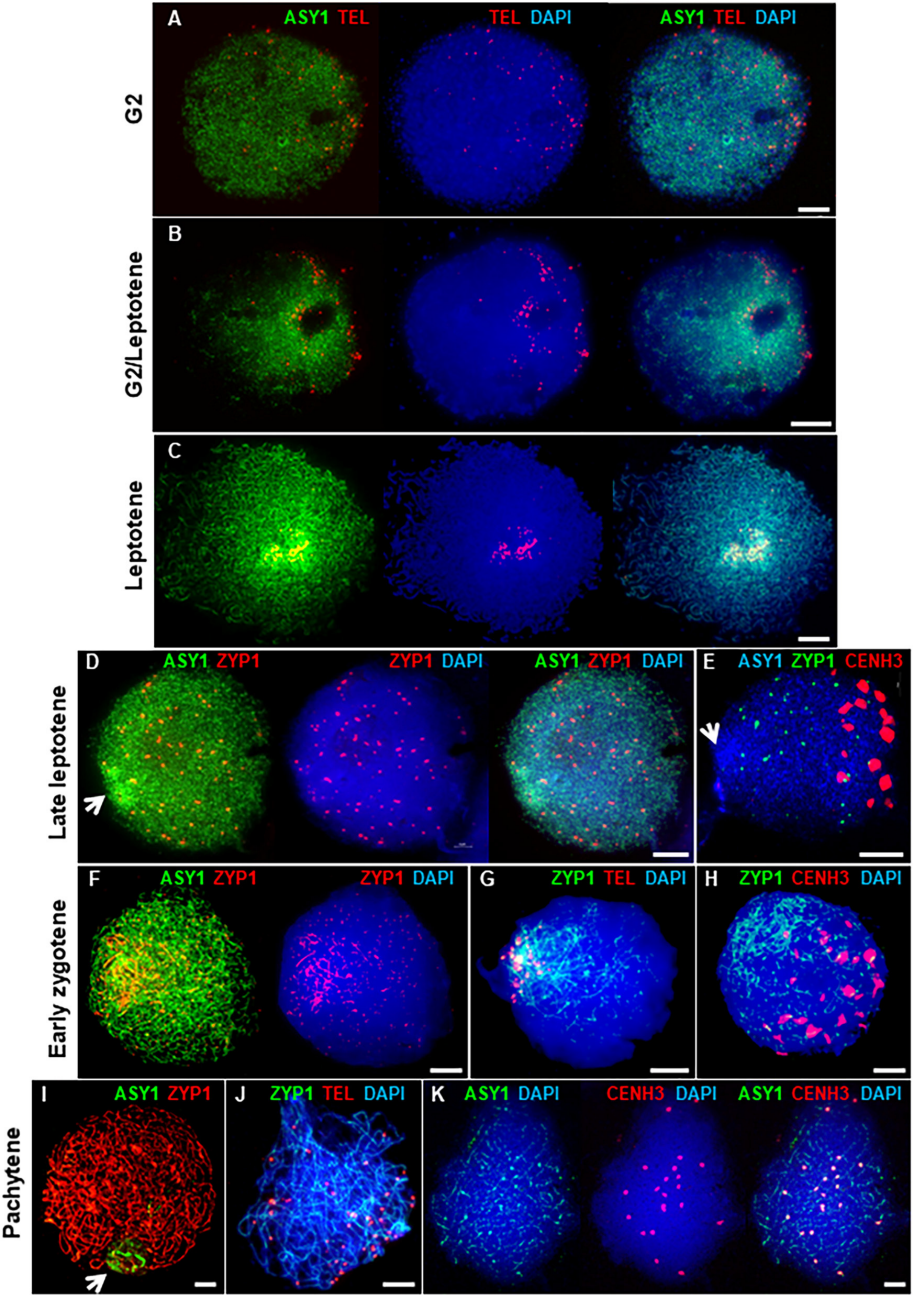
Axis and SC development in Cadenza. **(A–C)** Early prophase I showing bouquet formation. **(A)** G2: ASY1 foci and individual telomeres are widely distributed. **(B)** G2/Leptotene: telomeres begin to cluster and ASY1 linearization and enrichment begins in surrounding region. **(C)** Leptotene: telomeres are tightly clustered and ASY1 continues to linearize, appearing highly enriched in the bouquet region. **(D–K)** Synaptic stages. **(D,E)** ZYP1 forms presynaptic foci throughout the nucleus. Arrows mark ASY1 signal enrichment in the bouquet region. **(F–H)** Initial stretches of SC form in the distal regions. **(I–K)** Full synapsis. **(I)** ZYP1 forms a linear signal throughout the nucleus and ASY1 signal intensity is reduced apart from in the nucleolar region (arrow). **(J)** By pachytene telomere pairs are widely distributed. **(K)** Residual ASY1 signal is weakly enriched in paired centromeric regions. DNA is stained with DAPI (blue). For clarity, some images are shown in several color combinations. Bar = 10 μM.

The HORMA domain protein, ASY1, is a component of the meiotic chromosome axis essential for synapsis and wild type levels of COs (Caryl et al., 2000; Armstrong et al., 2002; Sanchez-Moran et al., 2007). In Arabidopsis and Brassica, ASY1 initially forms numerous foci throughout the nucleus in G2 (Armstrong et al., 2002; Sanchez-Moran et al., 2007). These then associate with the developing chromosome axis to form a linear signal along each pair of conjoined sister chromatids, which is characteristic of the leptotene sub-stage of prophase I. In PMCs of Cadenza, ASY1 also first appeared as weak foci throughout G2 nuclei (Figure 2A). At this stage, up to 84 widely dispersed telomeric FISH signals were observed (mean per nucleus = 69.4 ± 4.7; range = 49–84; *n* = 9), which tended to occupy one hemisphere of the nucleus. This is consistent with a pre-meiotic Rabl configuration of chromosomes, with telomeres and centromeres oriented to opposite hemispheres of the nucleus (Cowan et al., 2001; Sepsi et al., 2017). Telomere distribution then became more restricted, they began to cluster and ASY1 started to form short linear stretches around this region (Figure 2B). This was accompanied by an increase in ASY1 signal intensity in this region relative to the rest of the nucleus. As meiosis progressed, the number of telomere signals reduced as clustering continued, eventually forming the tight bouquet configuration which persisted during progressive linearization of the ASY1 signal throughout the more interstitial regions of the chromosomes (Figure 2C). By the time bouquet formation was complete, the ASY1 signal appeared highly enriched in the sub-telomeric (or distal) regions of chromosomes. This phenomenon was highly distinctive and could be used as a diagnostic marker for the bouquet region at this stage.

During zygotene, the SC forms between the aligned pairs of homologous chromosomes (Zickler and Kleckner, 1999; Page and Hawley, 2004). In Cadenza, development of the SC was monitored by immunolocalization of the transverse filament protein, ZYP1 (Higgins et al., 2005). In late leptotene, before any linear SC signal was observed, ZYP1 formed distinctive axis-associated foci distributed throughout the nucleus (mean no. of foci per nucleus = 83.9 ± 6.2; range = 28–128; *n* = 20). From now on, these will be referred to as “presynaptic” ZYP1 foci. They appeared after bouquet formation but before the ASY1 signal was completely continuous throughout the entire nucleus (Figure 2D) and dual localization with γH2A.X suggested they were located at a subset of DSB sites (see below and Supplementary Figure 2). At this stage the centromeric regions, marked by CENH3, were clustered in the opposite half of the nucleus to the distal regions and there was no particular colocalisation of the ZYP1 antibody with the centromeres (Figure 2E and Supplementary Figure 3). In early zygotene, ZYP1 began to form a linear signal in the distal regions. This signal continued to extend, coupled with the emergence of small foci and short stretches of ZYP1 in the interstitial regions, suggesting that SC formation initiates first in the sub-telomeric regions (Figure 2F). Later, some of the interstitial signals were observed to become more linear but by this time SC formation from the bouquet region was already well-established (Figure 2G). The telomere bouquet and the centromeres remained at opposite poles of the nucleus at least during the early stages of telomere-led synapsis (Figures 2G,H). As synapsis progressed the linear ZYP1 signals gradually extended, replacing the intense ASY1 signal as the chromosome axes were remodeled (Lambing et al., 2015). By pachytene, the linear ZYP1 signal extended throughout the nucleus and very little intense ASY1 signal remained apart from near the nucleolus (Figure 2I), consistent with observations in Arabidopsis that the nucleolar organizing regions do not undergo synapsis (Sims et al., 2019). At this stage, paired telomeres were no longer in the tight bouquet organization but were again widely dispersed (Figure 2J). Interestingly, although ASY1 signal was very weak when chromosomes were fully synapsed at pachytene, it was clearly enriched at the 21 pairs of centromeres (Figure 2K).

In summary, axis formation and synapsis were spatially asynchronous, beginning in the sub-telomeric (distal) regions of chromosomes before progressing to the interstitial and centromere proximal regions. During prophase I, the distal regions may therefore be considered to be in advance of the interstitial and proximal regions at any given time. For simplicity, we have taken the onset of leptotene and zygotene to be the start of ASY1 and ZYP1 signal linearization in the distal regions, respectively, although it will be appreciated that the interstitial/proximal regions will be lagging with respect to these stages.

### Chronology of Prophase I

**Figure 3:**
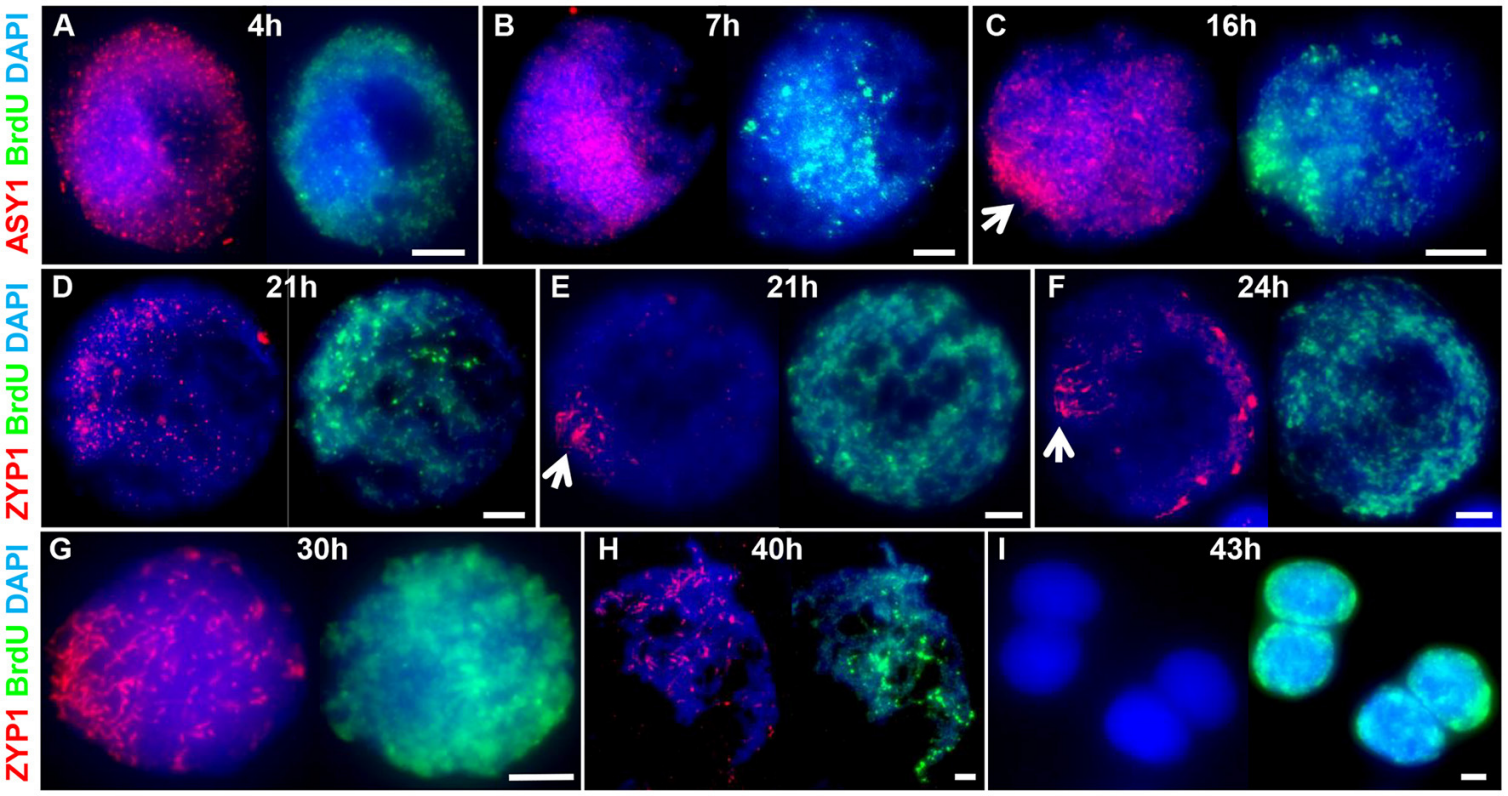
A meiotic time-course of Cadenza. BrdU was incorporated into newly synthesized DNA during pre-meiotic S-phase and samples were taken at set time points following injection and assessed for BrdU labeling. ASY1 and ZYP1 were used to determine meiotic stage. **(A)** Labeled G2 nuclei with ASY1 foci were observed at 4 h; **(B)** the onset of leptotene and start of bouquet formation at 7 h (marked by ASY1 linear stretches and polarization); **(C)** tight bouquet formation by 16 h (marked by highly polarized ASY1 signal - arrow); **(D)** ZYP1 pre-synaptic foci by 21 h; **(E,F)** onset of zygotene (marked by polarized ZYP1 linear stretches, arrow) by 21–24 h; **(G)** progression of synapsis through 30 h; **(H)** diplotene (desynapsis) at 40 h and **(I)** tetrads by 43 h. Note ZYP1 staining of centromere clusters at opposite pole to SC extension in early zygotene **(F)**. DNA is stained with DAPI. For clarity, several color combinations of images are shown. Bar = 5 μM.

To determine the chronology of the spatial asymmetry of axis formation and synapsis, we carried out an immunocytological time-course by monitoring ASY1 or ZYP1 localization in combination with 5-Bromo-2′-deoxyuridine (BrdU) labeling of replicating DNA in S-phase. BrdU delivery was based on a method developed for Arabidopsis (Armstrong et al., 2003) but involved direct injection of the growing plant as previously described for barley (Higgins et al., 2012; Ahn et al., 2020). BrdU was injected into the cavity above the developing spike (taken as Time 0 h) and followed by fixation of anthers at set time-points in order to determine the time taken from injection to landmark features of axis and SC development (as defined by ASY1 and ZYP1 localization, see above). For visualizing incorporated BrdU and ASY1/ZYP1 in PMCs we modified a previously described Arabidopsis immunolocalization protocol (Chelysheva et al., 2010). This enabled BrdU and ASY1/ZYP1 to be simultaneously labeled in fixed tissue chromosome spreads in one procedure. For each sample time, BrdU labeling of a subset of somatic anther nuclei provided a positive control for successful uptake into the anther, regardless of whether PMCs were labeled at that particular stage. For each time-point, we examined all meiotic stages in order to determine the latest meiotic stage to have incorporated BrdU, thus establishing a minimum time-frame for progression to that stage. For all time-points this assessment was based on observing a minimum of 20 BrdU labeled nuclei. For most time-points we also observed BrdU labeling of earlier stages (Supplementary Table 1). This variation was not surprising and could be due to several factors, including PMCs being at different stages of S-phase when exposed to BrdU, variation in the rate of meiotic progression between PMCs or differences in the time taken for BrdU to reach individual anthers. Figure 3 shows the latest BrdU labeled stage for each time-point (see also Supplementary Table 1). ASY1 was detected as foci by 4 h after BrdU injection (Figure 3A) and by 7 h had begun to form short linear stretches and appear polarized (Figure 3B). By 16 h the characteristic region of highly enriched ASY1 signal indicative of the bouquet had formed (Figure 3C). ZYP1 foci were apparent by 21 h (Figure 3D) and between 21 and 24 h short stretches of SC began to form in the distal regions (Figures 3E,F). By 24 h, after the appearance of linear stretches of SC, the ZYP1 antibody also appeared to strongly mark several large structures at the opposite pole of the nucleus (Figure 3F). These were similar in appearance and distribution to the CENH3 signals in Figures 2E,H and were thought to be clustered centromeres. Marking of these structures by the ZYP1 antibody appeared transient; it was not observed at the pre-synaptic ZYP1 foci stage, prior to linear SC formation (Figures 2D,E, 3D and Supplementary Figure 3), and became less obvious as synapsis progressed. Similar ZYP1 marking of the centromeric regions at specific stages of prophase I was reported in *T. aestivum* cv. Chinese Spring (Sepsi et al., 2017). As prophase I progressed, ZYP1 signal extension continued (Figure 3G) throughout the nucleus until full synapsis at pachytene and then was lost from chromosomes as they desynapsed at diplotene (by 40 h, Figure 3H). The subsequent division stages occurred rapidly, such that BrdU labeled tetrads were observed by 43 h (Figure 3I).

It was noticeable that during the initial stages of prophase I, the pattern of BrdU staining was consistent with it localizing to the distal regions of chromosomes, as marked by ASY1. Thus, when ASY1 foci appeared, distributed throughout the nucleus at 4 h (corresponding to the pre-bouquet stage when telomeres are widely dispersed, Figure 2A), BrdU localization was similarly widely distributed (Figure 3A). By 7 h, as ASY1 began to linearize and appear enriched within a restricted region of the nucleus (corresponding to the start of telomere clustering, Figure 2B), BrdU staining was similarly polarized (Figure 3B). Although not conclusive, this suggests that initial BrdU incorporation was in distal chromosome regions and implies that these chromosomal regions replicate first. By 16 h and in all subsequent stages ASY1 and BrdU staining were observed throughout the entire nucleus (Figures 3C-I), despite the bouquet configuration persisting until at least early zygotene (Figures 2G, 3G). This suggests that by 16 h, replication of interstitial and proximal DNA had taken place.

The meiotic time course is summarized in **Figure 7B** and shows that the minimum time for meiosis was 43 h. Leptotene occupied approximately 17 h with zygotene to diplotene taking 16 h. The remaining stages comprising diakinesis and the two meiotic divisions were completed relatively quickly, within 3 h.

### Initiation and Progression of Recombination Is Distally Biased

In Arabidopsis recombination progression during prophase I can be monitored by immunolocalization of key recombination proteins on meiotic chromosome spreads (Osman et al., 2011). Many of the antibodies developed during the course of Arabidopsis research have been useful for the analysis of other flowering plants, including barley (Higgins et al., 2012). We therefore anticipated that they would be successful in hexaploid wheat, particularly as wheat co-expression analysis has suggested that homoeologues of most meiotic genes are highly conserved and have not undergone sub/neo-functionalization (Alabdullah et al., 2019). This proved to be the case, so unless otherwise stated, the recombination antibodies we used were raised against Arabidopsis proteins.

**Figure 4:**
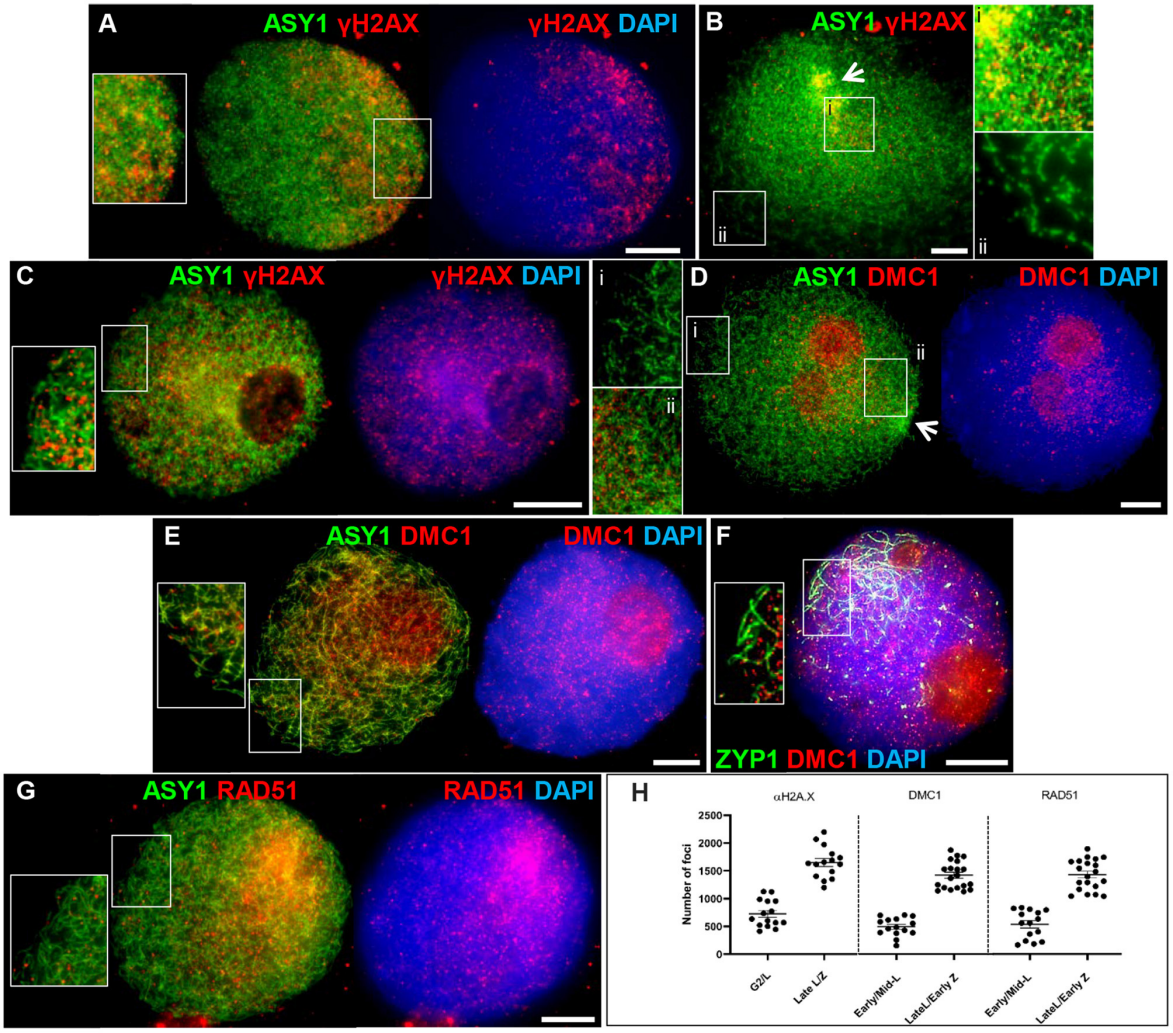
Initiation of recombination in Cadenza. **(A)** Late G2; **(B,D)** early leptotene; **(C)** late leptotene; **(E–G)** early zygotene. Recombination proteins were initially observed in the distal regions [γH2A.X **(A,B)** and DMC1 **(D)**], later occurring throughout the nucleus [γH2A.X **(C)**; DMC1 **(E,F)** and RAD51 **(G)**]. ASY1 and ZYP1 mark the axis and SC, respectively. Note enrichment of ASY1 signal in the bouquet region in early leptotene **(B,D)** (arrows) and polarized early SC extension (single frame only shown, for clarity) **(F)**. DNA is stained with DAPI. For clarity, some images are shown in several color combinations. **(H)** γH2A.X, DMC1, and RAD51 foci were counted when they were first observed and when they occurred throughout the nucleus. Numbers per PMC are shown with mean and standard error (SE) bars. Bar = 10 μM.

Meiotic recombination is initiated by the programmed formation of DSBs and is followed by the rapid phosphorylation of histone H2A.X around the break sites (Sanchez-Moran et al., 2007). We used an antibody specific to the phosphorylated form of HsH2A.X (γH2A.X) as a marker for DSBs in PMCs. γH2A.X foci were first observed enriched in one half of the nucleus in late G2, shortly after the appearance of ASY1 foci (between 4 and 7 h), and concomitant with the start of ASY1 signal linearization (Figure 4A). Subsequent bouquet formation confirmed that this region of enrichment corresponded to the distal chromosome regions where ASY1 linearization was most advanced; relatively few γH2A.X foci were observed in the more interstitial/proximal regions toward the nuclear periphery (Figure 4B). As ASY1 linearization progressed, the number of γH2A.X foci continued to increase, rising from a mean of 728 ± 63.1 per PMC (*n* = 15) around the time foci first appeared with up to 2,198 (mean = 1,651 ± 72.8, *n* =15) observed when ASY1 was fully linear in late leptotene/start of zygotene (Figure 4H). By this time foci were distributed throughout the nucleus (Figure 4C). These data are consistent with a study by Gardiner et al. (2019), which reported a mean of 2,133 DSBs per male meiosis at leptotene in hexaploid wheat.

Early stages of meiotic recombination are catalyzed by the coordinated activity of the strand exchange proteins, DMC1 and RAD51 (Neale and Keeney, 2006) and in Arabidopsis they are essential for CO formation and DSB repair, respectively (Couteau et al., 1999; Li et al., 2004). Wheat DMC1 foci localized to linear stretches of axis during leptotene as the ASY1 signal extended. Initial DMC1 localization was predominantly to the distal regions (marked by increased ASY1 staining) where axis development was most advanced; foci appeared in more interstitial/proximal regions at the opposite pole of the nucleus in late leptotene/early zygotene, when the ASY1 signal was linear throughout the nucleus (Figures 4D,E). Foci were counted in early-mid leptotene when they were first detected (mean per PMC = 494 ± 42.8, *n* = 15) and in late leptotene/early zygotene (maximum = 1,875, mean = 1,421 ± 55.0, *n* = 20), indicating an accumulation of foci as the chromosome axes progressively linearized (Figure 4H). For both counts, the large range reflects the highly dynamic nature of the process. Foci remained prominent throughout the nucleus during the early stages of zygotene where they marked early stretches of SC as the axes began to synapse (Figure 4F). During the later stages of prophase I foci numbers decreased (mid-late zygotene mean = 314 ± 68.3, *n* = 5) and signal was mostly gone by pachytene (Supplementary Figure 4A). These data are consistent with DMC1 counts reported for *T. aestivum* cv. Renan (Benyahya et al., 2020). The DMC1 antibody also prominently stained the nucleoli. This phenomenon has been observed in other plant species and for other antibodies and it has been suggested that the nucleolus may act as a reservoir for sequestering meiotic proteins, as it does for cell cycle proteins (Visintin and Amon, 2000; Jackson et al., 2006; Vignard et al., 2007; Higgins et al., 2012). Alternatively, non-specific staining of the nucleoli may be occurring due to its high protein content. RAD51 scored at similar stages to DMC1 showed similar loading dynamics (early-mid leptotene, mean = 537 ± 65.6, *n* = 15; late leptotene/early zygotene, maximum = 1,897, mean = 1,431 ± 61.2, *n* = 20; mid-late zygotene, mean = 259 ± 75.9, *n* = 5) (Figures 4G,H and Supplementary Figure 4B).

**Figure 5:**
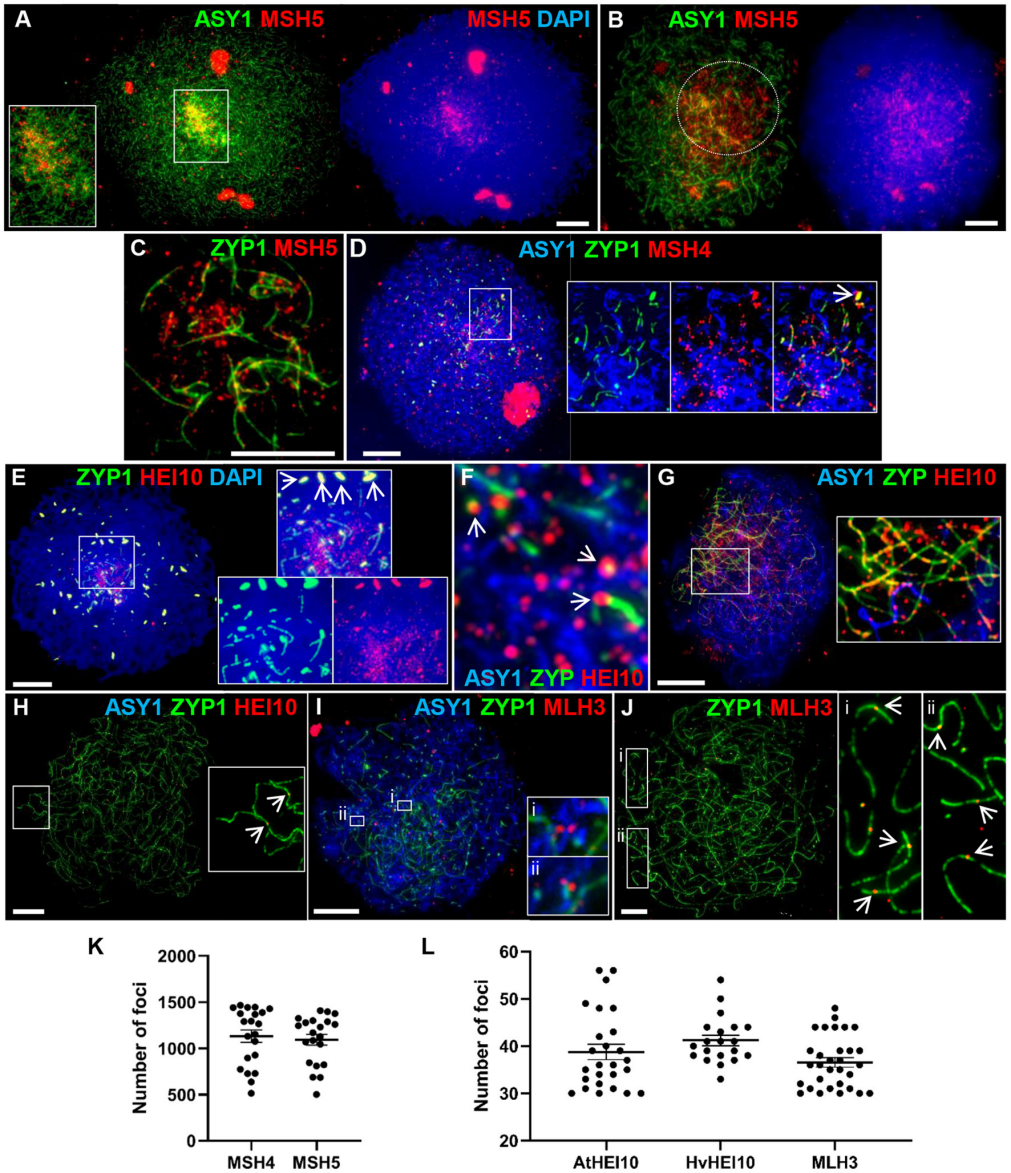
Recombination progression in Cadenza showing MSH4/5, HEI10 and MLH3 localization. Axes and SC were marked by ASY1 and ZYP1, respectively. **(A)** Late leptotene: initial MSH5 foci were predominantly in distal regions, marked by higher intensity ASY1 signal. **(B)** Early zygotene: MSH5 localized throughout the nucleus but foci appeared more numerous in distal regions where ASY1 signal was depleted as chromosomes synapsed (marked by dotted circle). **(C)** Single frame detail of early zygotene nucleus showing localization of MSH5 foci to initial stretches of SC and to (unmarked) unsynapsed axes. **(D)** Start of zygotene: triple immunostaining showing MSH4 foci localized to unsynapsed axes, ZYP1 foci (inset, arrow) and early stretches of SC. **(E)** Start of zygotene showing numerous HEI10 foci surrounding initial stretches of SC in the distal regions and more prominent foci associated with SC ends (inset). HEI10 also colocalized with bright ZYP1 foci throughout the nucleus (main image and inset, arrows). **(F)** Detail of triple immunostained nucleus confirming HEI10 localization to unsynapsed axes and association of prominent foci with nascent SC (arrows). **(G)** Progression of zygotene showing HEI10 foci localized along the extending SC. Regularly spaced stretches of foci were observed along the SC and unsynapsed axes (inset). **(H)** Pachytene nucleus showing remaining HEI10 foci on SC. Inset shows two distal foci (arrows). **(I)** MLH3 localization in early zygotene. Insets show examples of foci pairs apparently flanking the axis or nascent SC. **(J)** At pachytene the number and position of prominent MLH3 foci was consistent with their marking CO sites. Insets show examples of near-distal foci (arrows). **(K)** MSH4 and MSH5 foci were counted as they accumulated in late leptotene/early zygotene. **(L)** Foci marked by the AtHEI10 or HvMLH3 antibodies were counted in late pachytene, foci marked by the HvHEI10 antibody were counted in late pachytene/diplotene. Numbers of foci per PMC are shown with mean and SE bars. Bar = 10 μM.

The meiosis-specific MutS homologs, MSH4 and MSH5, function as a heterodimer and bind and stabilize double Holliday Junction (dHJ) recombination intermediates (Snowden et al., 2004). In Arabidopsis and tetraploid wheat, MSH4 and MSH5 are essential for the formation of interference-sensitive Class I COs that account for ~85% of COs (Higgins et al., 2004; Desjardins et al., 2020). In Cadenza MSH5 was first observed as foci along linear stretches of axis in late leptotene, as well as staining the nucleoli. As with DMC1 and RAD51, initial loading was predominantly distal where axis development was most advanced (Figure 5A). Foci rapidly increased in number and at early zygotene foci were present throughout the nucleus, yet still appeared more numerous in the distal regions where chromosomes began to synapse (evidenced by depletion in ASY1 signal) (Figure 5B). As well as localizing to unsynapsed axes, dual staining with ZYP1 at this stage confirmed that MSH5 localizes to early stretches of SC (Figure 5C). MSH4 also localized as foci during late leptotene/early zygotene where it marked unsynapsed linear axis, ZYP1 foci (see above) and nascent stretches of SC (Figure 5D). Staining of the nucleolus was also observed. Counts of MSH4 and MSH5 foci as they accumulated in late leptotene/early zygotene ranged from 513 to 1,465 (mean = 1,125) and 501 to 1,409 (mean = 1,096) per PMC, respectively (*n* = 20) (Figure 5K). From mid-zygotene onwards MSH4 and MSH5 foci gradually declined in number so that by pachytene only a few remained associated with the SC (MSH4 mean = 16 ± 2.0; MSH5 mean = 14 ± 3.1; *n* = 6) (Supplementary Figures 5A,B).

HEI10 is a member of the Zip3/HEI10 family of proteins thought to possess SUMO/ubiquitin E3 ligase activity (Chelysheva et al., 2012; Wang et al., 2012). Zip3/HEI10 marks Class I CO sites and in *A. thaliana* and *Sordaria macrospora* HEI10 foci at these sites have been shown to emerge from a much larger population of smaller axis-associated foci in early/mid prophase I (Chelysheva et al., 2012; De Muyt et al., 2014). In Cadenza HEI10 localization was investigated using antibodies against the Arabidopsis and barley proteins (Lambing et al., 2015; Desjardins et al., 2020). HEI10 was first detected in late leptotene where it colocalized with the presynaptic ZYP1 foci (see above) (Supplementary Figure 6A). Colocalization at bright ZYP1 foci was still apparent at the start of zygotene when the SC began to extend in the distal regions (Figure 5E). In addition, the ends of some of the linear ZYP1 stretches appeared to be associated with prominent HEI10 foci and numerous smaller HEI10 foci were observed in the chromatin immediately surrounding this region (Figure 5E). Triple localization of HEI10, ASY1, and ZYP1 at this stage indicated that HEI10 foci localize to unsynapsed axes (marked by ASY1) and confirmed the association of prominent HEI10 foci with nascent SC (Figure 5F). As the SC extended, HEI10 foci localized all along its length and stretches of regularly spaced foci could be observed on both unsynapsed axes and linear SC (Figure 5G). In late prophase I, HEI10 foci gradually became depleted from chromosomes leaving a sub-population of prominent foci. By pachytene, it was clear that many of the remaining foci were near to chromosome ends, consistent with marking CO sites (Figure 5H). In diplotene, foci marked by the AtHEI10 antibody quickly disappeared from chromosomes as they began to desynapse. However, foci marked by the HvHEI10 antibody remained detectable during early diplotene where they associated with residual stretches of ZYP1 staining (Supplementary Figure 6B). The mean number of prominent SC-associated foci per PMC at late pachytene was 38.8 ± 1.6 per PMC (*n* = 26) using the AtHEI10 antibody and 41.2 ± 1.1 (*n* = 20) with the HvHEI10 antibody (counted at late pachytene/diplotene) (Figure 5L).

The MutL homologs MLH1 and MLH3 act as a heterodimer to ensure that dHJs are resolved as COs rather than non-COs (Hunter and Borts, 1997; Wang et al., 1999; Cannavo et al., 2020; Kulkarni et al., 2020). Immunogold labeling has shown that the two proteins provide a reliable marker for Class I COs at pachytene (Moens et al., 2002; Lhuissier et al., 2007), and they have been routinely used for this purpose in a variety of organisms including plants (Jackson et al., 2006; Chelysheva et al., 2010; Phillips et al., 2013). We have investigated MLH3 localization in Cadenza using an antibody raised against the barley protein (Phillips et al., 2013). MLH3 foci are present in nuclei during the early stages of zygotene, but localization at this stage is not specific to the developing SC and many associate with the unsynapsed axis (Figure 5I). Interestingly, several examples of pairs of foci apparently flanking the axis or nascent SC were observed (Figure 5I detail). By pachytene, when the chromosomes were fully synapsed, localization of prominent MLH3 foci was largely confined to the linear SC signal (Figure 5J), with foci frequently located near the chromosome ends, consistent with marking CO sites (Figure 5J detail). The mean number of SC-associated MLH3 foci at pachytene was 36.6 ± 1.0 (*n* = 31) (Figure 5L), falling to 20.9 ± 2.6 (*n* = 14) as they became depleted from the chromosomes in early diplotene.

### H3K4me3, H3K27me3 and H3K9me3 Histone Marks Are Enriched in the Distal Regions in Early Prophase I

**Figure 6:**
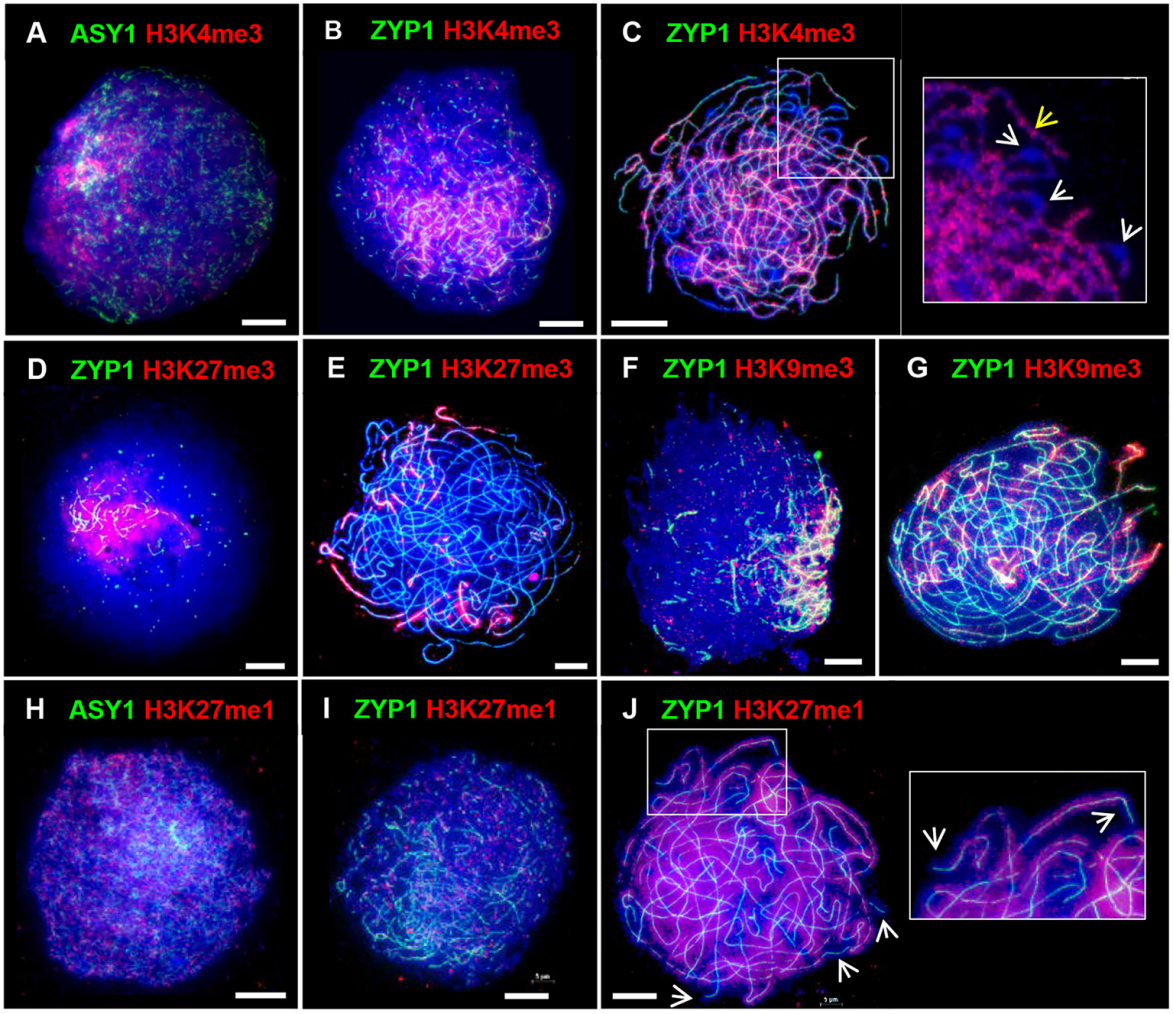
Distribution of histone marks in Cadenza prophase I. ASY1 is used to mark the axes in leptotene; ZYP1 the SC from zygotene onwards. Gene-associated histone marks: **(A–C)** H3K4me3 at leptotene, early zygotene and pachytene, respectively - detail at pachytene shows discrete bands of H3K4me3 staining along chromosomes (yellow arrow) and likely paired centromeric regions devoid of staining (white arrows); **(D,E)** H3K27me3, a repressive mark, at early zygotene and pachytene, respectively; **(F,G)** H3K9me3 at early zygotene and pachytene, respectively. **(H–J)** H3K27me1, a marker of heterochromatin and TEs, at leptotene, zygotene and pachytene, respectively – detail and arrows at pachytene show absence of staining at chromosome ends. DNA is stained with DAPI (blue). Bar = 10 μM.

In barley the spatio-temporal asymmetry of meiotic progression and eventual chiasma localization was potentially associated with the distal distribution of early-replicating euchromatin (Higgins et al., 2012). We were therefore interested in investigating the distribution of chromatin histone modifications in PMCs of Cadenza. H3K4me3, a marker of active genes, promotes recombination in budding yeast and has been shown to be associated with recombination sites in a range of other organisms, including plants (Borde et al., 2009; Liu et al., 2009; Choi et al., 2013; Shilo et al., 2015; Adam et al., 2018). In Cadenza H3K4me3 localized to chromatin throughout prophase I and in leptotene and early zygotene appeared enriched in the distal chromosome regions (Figures 6A,B). Distal enrichment was less obvious at pachytene when H3K4me3 was more widely distributed, often forming bands of increased signal intensity along chromosomes (Figure 6C). At this stage H3K4me3 staining was noticeably absent from bulbous regions of DAPI-stained chromatin, which were likely sites of heterochromatin and paired centromeres (Figure 6C). H3K27me3 is associated with repression of gene transcription and marks facultative heterochromatin (Ramírez-González et al., 2018; Concia et al., 2020). This mark was highly enriched in distal regions during prophase I (Figures 6D,E), and in pachytene appeared to be preferentially marking chromosome ends (Figure 6E). Interestingly, H3K9me3 was also highly enriched in the distal regions in prophase I (Figures 6F,G). Although this is a marker of constitutive heterochromatin in mammals, in Arabidopsis it marks euchromatin and is reportedly associated with genes (Naumann et al., 2005; Roudier et al., 2011). Similar H3K9me3 distal enrichment was observed in barley PMCs in prophase I (Higgins et al., 2012). Finally, H3K27me1, a marker of heterochromatin and transposable elements (TEs) in plants, including wheat (Jacob and Michaels, 2009; Concia et al., 2020), showed generalized chromatin staining throughout the nucleus during prophase I (Figures 6H–J), and by pachytene it became evident that chromosomes were fairly evenly stained apart from near their ends (Figure 6J). In summary, the histone marks H3K4me3, H3K27me3, and H3K9me3 were enriched in the gene-rich distal regions in early prophase I.

### Chromatin Exhibits Contraction/Expansion Cycles During Prophase I

**Figure 7:**
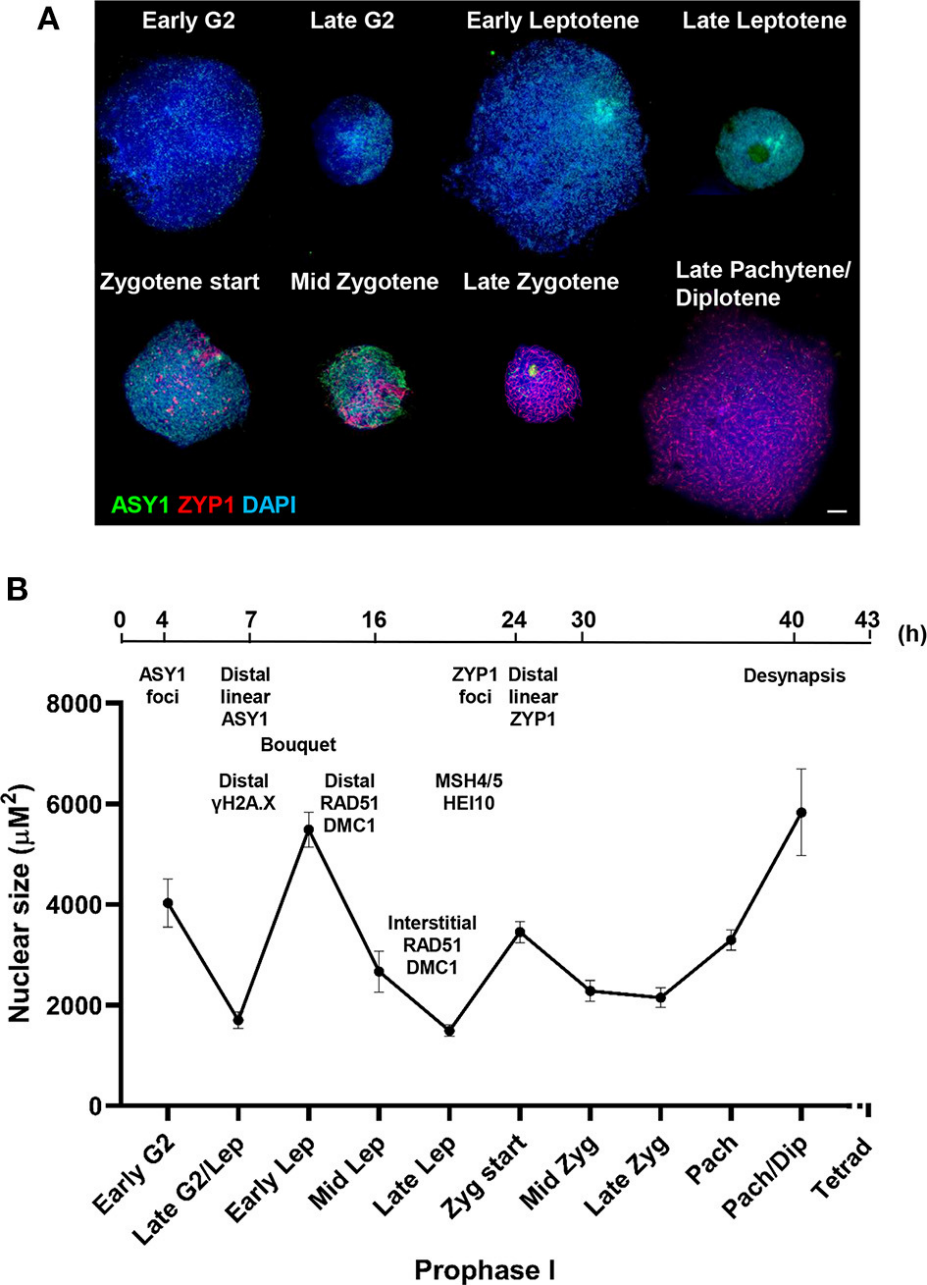
Chromatin contraction/expansion cycles in Cadenza prophase I. **(A)** Immunolocalization of ASY1 (green) and ZYP1 (red) showing that initial distal meiotic events coincide with nuclear expansion phases. DNA is stained with DAPI (blue). Bar = 10 μM. **(B)** Mean nuclear size of PMCs at key stages of prophase I, as defined by ASY1 and ZYP1 staining. Bars represent standard error. A time-line of key events is included, based on cross-referencing ASY1/ZYP1 staining with a BrdU time course and refers to the “leading edge” of meiotic progression (i.e., not all PMCs within a sample may reach a particular stage in the time indicated). Times indicate hours after BrdU injection and the time-line is not to scale.

A mechanical stress model of chromosome function has been developed based on the observation that eukaryotic mitotic and meiotic programs comprise global cycles of chromatin expansion and contraction (Kleckner et al., 2004). During meiotic prophase I, chromatin undergoes successive cycles which correlate with well-defined cytological stages and are proposed to coordinate four temporally distinct steps leading to CO formation: DSB formation; strand exchange; dHJ formation and dHJ resolution. Analysis of the meiotic program in barley showed that meiotic progression in the distal chromosome regions is coordinated with the expansion/contraction cycles (Higgins et al., 2012), so we were interested in whether a similar relationship exists in wheat. Cell walls of PMCs were digested so that nuclei occupied an in-solution envelope volume as determined by their chromatin state (Kleckner et al., 2004). As described in Higgins et al. (2012), changes in envelope volume were assessed by measuring the size of nuclei at specific stages, defined by ASY1 and ZYP1 localization patterns (Figures 7A,B). In early G2, when ASY1 first appeared as weak foci, nuclei were relatively large (mean = 4,029 μm^2^ ± 480.3, *n* = 12). By late G2, when ASY1 began to exhibit loosely polarized signal enrichment and initiate short linear stretches, nuclear size had significantly reduced (mean = 1,703 μm^2^ ± 161.3, *n* = 14, *P* < 0.0001). However, by the time ASY1 linearization was clearly established in the distal regions in early leptotene, nuclear size had increased again (mean = 5,489 μm^2^ ± 344.9, *n* = 41, *P* < 0.0001). A second significant reduction in nuclear size had occurred by late leptotene when ASY1 signal was almost continuous (mean = 1,493 μm^2^ ± 112.8, *n* = 16, *P* < 0.0001). This was followed by an increase at the start of zygotene when linear stretches of ZYP1 staining began to appear in the distal regions (mean = 3,454 μm^2^ ± 208.7, *n* = 26, *P* < 0.0001). Nuclei had undergone a third significant contraction by mid-zygotene when SC polymerisation was ~50% complete (mean = 2,284 μm^2^ ± 206.4, *n* = 20, *P* < 0.001), remaining like this until full synapsis at the end of zygotene (mean = 2,150 μm^2^ ± 191.9, *n* = 21, *P* = 0.64). Finally, by late pachytene/early diplotene, when ZYP1 began to disappear from chromosomes, nuclei had increased in size again (mean = 5,831 μm^2^ ± 861.2, *n* = 7, *P* < 0.0001). These data therefore support the existence of chromatin contraction/expansion cycles in wheat similar to those in barley and other species (Kleckner et al., 2004; Higgins et al., 2012). Furthermore, as in barley, the timing of key events in the distal regions, such as RAD51/DMC1 localization, appear to coincide with periods of relative chromatin expansion.

## Discussion

Advances in wheat genomics and genome engineering present new opportunities to manipulate CO frequency and distribution to realize the potential of genetic variation for crop improvement (Adamski et al., 2020; Taagen et al., 2020). To provide a cytogenetic reference framework for CO modification initiatives and functional studies of meiotic recombination, we performed a detailed cytological analysis of recombination progression in the hexaploid spring wheat variety, Cadenza. We showed that there is a spatio-temporal bias in the initiation and progression of recombination that mirrors the tendency of chiasmata/COs to occur in the gene-dense distal regions of the chromosomes and is reflected in the distribution of gene-associated histone marks in the genome. We established a time-frame for the duration of meiosis and confirmed that wheat chromatin undergoes cycles of contraction and expansion during prophase I as previously observed in barley and other species (Kleckner et al., 2004; Higgins et al., 2012). During the course of this study, we also noted interesting aspects of ASY1 and ZYP1 protein localization during the meiotic program.

### Chiasmata Occur Predominantly in Gene-Dense Distal Chromosomal Regions

Historically, the large chromosomes of wheat and other cereals have made them ideal candidates for cytogenetics studies so the tendency of chiasmata/COs to occur in the distal regions of chromosomes has long been known. More recently, wheat studies involving genetic mapping and whole-genome sequencing have provided fine-scale confirmation of this bias (Choulet et al., 2014; Darrier et al., 2017; Jordan et al., 2018; Gardiner et al., 2019). Formal quantification in Cadenza revealed a total mean chiasma frequency of 41.8 per PMC and confirmed that 88% of all chiasmata were in the terminal/sub-terminal regions of the chromosomes. Even so, the presence of chiasmata in the interstitial/proximal regions, albeit relatively infrequent, does at least support the feasibility of targeting COs to these regions. It would be interesting to carry out further analysis using genomic *in situ* hybridization (GISH) and chromosome-specific FISH probes to determine whether interstitial/proximal chiasmata favor particular sub-genomes, chromosomes or chromosome regions.

During early prophase I H3K4me3, a marker of euchromatin, and H3K27me3, a marker of facultative heterochromatin were enriched in the distal regions. H3K27me3 distal enrichment has also been reported in wheat somatic nuclei (Concia et al., 2020; Liu et al., 2020). H3K9me3 also showed clear distal enrichment in prophase I, similar to its distribution in barley meiosis (Higgins et al., 2012). Although H3K9me3 marks constitutive heterochromatin in mammals, the dimethylated form is thought to be the major mark of heterochromatin in Arabidopsis (Jackson et al., 2004) and H3K9me3 is reportedly associated with euchromatin and genes (Naumann et al., 2005; Roudier et al., 2011; Xu and Jiang, 2020). The localization patterns of H3K4me3, H3K27me3, and H3K9me3 are therefore consistent with the high gene-density at distal regions previously reported by Choulet et al. (2014). On the other hand, distribution of H3K27me1, a marker of heterochromatin, was relatively homogeneous throughout most of the chromatin with the exception of the distal regions, where signal was absent. Thus, chiasmata/CO distribution broadly coincides with gene-rich DNA in Cadenza PMCs as it does in other organisms. However, in plants, any direct relationship between CO distribution and gene density or specific histone mark remains to be established. Despite the importance of gene density in influencing global distribution patterns of recombination, the factors that shape recombination are complex, involving multiple regulatory layers and fine-tuning at the local level (Dluzewska et al., 2018; Fayos et al., 2019). This is illustrated by fine-scale mapping of *A. thaliana* floral tissue which revealed a complex relationship between H3K4me3 levels and DSBs (Choi et al., 2018). Thus, H3K4me3 was enriched in proximity to SPO11-1-oligo hotspots at gene 5′ ends but hotspots also occurred at the 3′ end of genes where H3K4me3 was less abundant (Choi et al., 2018).

Chiasma frequency provides a cytological estimate of the total number of COs per nucleus while the recombination proteins HEI10 and MLH3 are markers of interference-sensitive (Class I) COs in late prophase I (Jackson et al., 2006; Chelysheva et al., 2012; Phillips et al., 2013). The proportion of Class I COs in hexaploid wheat is yet to be determined. However, in *A. thaliana*, rice (*Oryza sativa*), tomato (*Solanum lycopersicum*), oil seed rape (*Brassica napus*) and allotetraploid durum wheat (*Triticum turgidum subsp. durum*) they account for ~85% of all COs suggesting that this proportion is widely conserved among plants (Higgins et al., 2004, 2008b; Luo et al., 2013; Anderson et al., 2014; Wang et al., 2016; Gonzalo et al., 2019; Desjardins et al., 2020). Assuming that 85% of COs are Class I in hexaploid wheat, we might expect them to account for ~35.5 COs per PMC based on total chiasmata frequency. Our observed estimates of 36.6, 38.8, and 41.2 from the HvMLH3, AtHEI10, and HvHEI10 antibodies, respectively, therefore appear reasonable. The slightly higher estimates observed with the HEI10 antibodies may simply reflect the polarized nature of wheat prophase I progression whereby disappearance of “early” HEI10 foci from interstitial regions lags behind distal regions, thus emphasizing the need to score this marker as late in prophase I as possible. Alternatively, the possibility that HEI10 has additional roles in wheat cannot be ruled out. It should also be pointed out that chiasma counts may underestimate CO frequency due to COs which are very close together being difficult to resolve at the cytological level.

In addition to the MLH3 foci that marked COs at pachytene, we also observed pairs of foci flanking the axis and nascent stretches of SC during early zygotene. CO formation is dependent on the MutLγ complex, which comprises MLH3 and the MutL mismatch repair protein MLH1 (Cannavo et al., 2020; Kulkarni et al., 2020). MLH1 has also been implicated in the resolution of chromosome interlocks during zygotene (Storlazzi et al., 2010). Hence it is possible that the MutLγ complex itself has a role in interlock resolution and the pairs of MLH3 foci observed during early zygotene in wheat reflect this activity.

### Recombination Initiation and Progression Exhibit a Spatio-Temporal Bias

In barley the spatio-temporal pattern of meiotic recombination is established during pre-meiotic S-phase whereby distal euchromatin-rich DNA regions are replicated first (0–4 h), followed by interstitial DNA (by 6 h) and finally proximal heterochromatin (by 13 h) (Higgins et al., 2012). Subsequent studies in budding yeast established a clear mechanistic link between the timing of DNA replication and downstream recombination initiation (Murakami and Keeney, 2014). In our study the distribution of early BrdU staining, particularly obvious during bouquet formation, is compelling evidence that DNA replication in the distal regions also occurs earlier than in interstitial/proximal regions in hexaploid wheat. Further support for this comes from an investigation of the dynamics of DNA replication in pre-meiosis and meiosis of *T. aestivum* cv. Chinese Spring using flow-cytometry which showed that replication in PMCs continues beyond the stage of bouquet formation and chromosome pairing in the distal regions (Rey and Prieto, 2014).

Immunolocalization of meiotic chromosome axis, SC and recombination proteins during early prophase I revealed a distal bias. Initial linearization of the ASY1 (axis) signal occurred predominantly, although not exclusively, in the sub-telomeric regions. Similarly subsequent SC extension, marked by linear ZYP1, also began in the distal regions. Sub-telomeric initiation of synapsis was previously described in *T. aestivum* cv. Chinese Spring (Sepsi et al., 2017). Here we have used BrdU labeling of DNA to determine a precise chronology for axis and SC development during prophase I. Dual localization of the recombination proteins with ASY1 and ZYP1 then allowed the initiation and progression of recombination to be indirectly anchored to the BrdU time-line. This confirmed that meiotic events in the distal regions preceded those in the interstitial/proximal regions by several hours and initiation of recombination, marked by γH2A.X foci, began in the distal regions before the meiotic axis was fully linear in the interstitial/proximal regions. As prophase I progressed this bias was maintained and the first appearances of RAD51, DMC1, MSH4/5, and HEI10 foci were similarly polarized.

At the leptotene/zygotene transition numerous DSBs were detected throughout the nucleus. At this stage up to 130 axis-associated ZYP1 foci were observed throughout the nucleus prior to the appearance of linear SC. Colocalization with γH2A.X, MSH4, and HEI10 suggested that the ZYP1 foci were located at a sub-set of recombination interactions raising the question as to their significance. In budding yeast synapsis initiation sites correspond to CO designated recombination intermediates (Fung et al., 2004). However, in species with larger chromosomes, such as some fungi, plants, insects, and animals, in addition to SC nucleations which correspond to designated COs, there are additional SC nucleations at recombination sites that will not become COs (Zhang et al., 2014). For example, in *Sordaria macrosporum* there are 40 or so SC nucleations about half of which correspond to CO sites and in barley SC initiates at about 55 sites and CO estimates range from 13.6 to 22.7 (Li et al., 2010; Higgins et al., 2012; Phillips et al., 2013; Zhang et al., 2014). These additional SC nucleation sites are thought to aid efficient synapsis (Zickler and Kleckner, 1999). Studies in *S. macrosporum* have led to the proposal that in fungi, plants and mammals, a single round of interference acting on early recombination intermediates gives rise to an evenly patterned array of synapsis initiation sites, including the subset which are CO designated (Zhang et al., 2014). This may account for the ZYP1 foci in wheat. However, further study will be needed to determine if this is the case not least because SC extension is first apparent in the distal regions prior to other chromosomal regions. Also, as the SC polymerises during early zygotene the ZYP1 foci become less obvious with the emergence of short stretches of SC and smaller foci throughout the nuclear volume.

An alternative explanation for the pre-synaptic ZYP1 foci is suggested by a study of the role of the 26*S* proteasome in meiotic chromosome pairing and recombination in budding yeast (Ahuja et al., 2017). In early prophase I, prior to the DSB-induced homology search, non-homologous chromosomal interactions could become stabilized by the promiscuous association of SC proteins, including Zip1. Recruitment of the proteasome served to displace the SC proteins, restricting their localization to centromeres and allowing normal homologous pairing and a coordinated transition to SC assembly. Furthermore, proteolytic core and regulatory particles were recruited to the chromosomes by Zip1 and Zip3, in an evolutionarily conserved manner (Ahuja et al., 2017). It therefore seems possible that the presynaptic ZYP1 foci we observed in wheat represent similar promiscuous non-homologous interactions and even that ZYP1 (and possibly HEI10) has an analogous role to that in budding yeast in recruitment of the proteasome to chromosomes.

### Factors Influencing Distal Bias of COs – Considerations

This study has revealed that the wheat meiotic program shares a number of similarities with barley meiosis: spatio-temporal asymmetry of axis and SC development and recombination initiation/progression; likely early-replicating distal euchromatin, nuclear contraction/expansion cycles at specific stages during prophase I and the overall duration of meiosis (minimum time to tetrad stage ~43 h) (Higgins et al., 2012). The timing of specific events within the meiotic programs was also very similar: first appearance of ASY1 foci (by 4 h in both species); elongation of ASY1 signal to form short linear stretches (by 7 h in wheat, 6 h in barley), first linear stretches of SC in the distal regions (by 24 h in wheat, 25 h in barley) and desynapsis (by 40 h in wheat, 39 h in barley). This was perhaps surprising given that bread wheat is a hexaploid so has an overall genome size three times that of diploid barley (~16.5 and ~5.3 Gb, respectively). However, their chromosomes are of a similar physical size (IWGSC, 2018; https://www.barleygenome.org.uk). Our estimate of 43 h for the duration of meiosis in Cadenza was carried out under strictly controlled growth conditions, including a temperature of 20°C (see Materials and Methods for details) and is considerably longer than the 24 h previously reported for cv. Chinese Spring grown at 20°C (Bennett et al., 1971). This earlier study was carried out before the routine use of immunocytology to study meiosis and employed sampling methods and the use of tritiated thymidine to label DNA. Interestingly, in a later publication, the same author reported that at a temperature of 15°C the duration of meiosis in Chinese Spring was 43 h; identical to our Cadenza estimate (Bennett, 1977). It therefore remains to be established whether the observed differences in timing at the reported temperature of 20°C reflect genuine varietal differences or differing environmental conditions and/or methodology.

Studies in budding yeast indicate that the CO/NCO decision is made early in the meiotic program and likely precedes stable strand exchange (Bishop and Zickler, 2004; Börner et al., 2004). At early leptotene in wheat, DSBs and early recombination pathway proteins were predominantly detected in the distal euchromatic chromosome regions. At this stage some appeared elsewhere in the chromatin, albeit relatively infrequently before increasing in abundance as prophase I progressed. By late leptotene DSBs were present throughout the nucleus. Since COs are rare in interstitial/proximal regions it seems that generally these early “non-distal” DSBs do not progress to form COs. The reason for this is not fully clear. One possibility is that in the initial stages, the high levels of DSBs in the distal regions (relative to the non-distal), combined with telomere anchoring of the chromosomes to the nuclear membrane, may promote homolog engagement here before the more interstitial/proximal regions have received sufficient breaks to achieve this. Additionally, it is worth considering that centromere dynamics during prophase I may also influence stable homolog engagement. In early leptotene, as the axis begins to linearize and shortly before formation of the telomere bouquet, centromeres of cv. Chinese Spring cluster into ~10 groups at the opposite pole of the nucleus (Sepsi et al., 2017). These clusters remain until early zygotene (this study, Sepsi et al., 2017). Coincident with the start of SC extension in the sub-telomeric regions, centromeres begin to be released from the clusters in a gradual, progressive manner with homologous centromeres released individually (and not necessarily from the same cluster) and it is suggested that this orderly release of centromeres may facilitate homologous pairing by restricting release to those undergoing pairing (Sepsi et al., 2017). This strategy would help to overcome the challenge of pairing large chromosomes whilst avoiding homoeologous pairing and minimizing the risk of chromosome interlocks. Based on this model, interstitial/proximal regions of chromosomes might be physically prevented from engaging with their homolog until after their distal regions have synapsed. Interestingly, ZYP1 colocalizes with the centromeric clusters in early zygotene (this study, Sepsi et al., 2017). Presynaptic centromeric localization of the SC proteins Zip1 and C(3)G have also been observed in budding yeast and Drosophila, respectively, where they are required for centromeric associations (Tsubouchi and Roeder, 2005; Takeo et al., 2011; Tanneti et al., 2011), suggesting a possible role for ZYP1 in the regulation of wheat centromere dynamics (for detailed discussion see Sepsi et al., 2017).

A consequence of early homolog engagement in distal regions might be that CO designation is similarly spatially-biased and less likely to occur in interstitial/proximal regions as CO interference will disfavor COs in adjacent chromosomal regions. That said, analysis of the distribution of class I COs based on MLH3 foci along barley chromosomes 2H and 3H revealed respective mean inter-focus distances of 38.5 and 42.6% of total SC length but of these, 38 and 34%, respectively, were <20% apart (Phillips et al., 2013). This implies that CO interference may not entirely account for the deficit in interstitial/proximal COs in the grasses. In barley it has been proposed that the coordination of the appearance of the recombination foci on the chromosomes with the chromatin contraction/expansion cycles may be a contributory factor to the distal bias (Higgins et al., 2012). Chromatin organization is also likely important, indeed in barley MLH3 inter-focus distances were found to be increased across the centromeric regions suggesting an influence of the pericentromeric heterochromatin (Phillips et al., 2013). It seems possible that CO distribution is similarly influenced in wheat.

The relationship between the meiotic chromosome axis/SC and recombination is intimate and complex (Zickler and Kleckner, 1999). ASY1 has long been recognized as a key component in the coordination of these events in plant genomes (Caryl et al., 2000; Sanchez-Moran et al., 2007; Osman et al., 2011). ASY1 is a core axis component, necessary for wild type CO levels, which additionally acts in a dosage-dependent manner to influence the distribution of COs along chromosomes (Lambing et al., 2020b). Chromatin-immunoprecipitation (ChIP) revealed a gradient of ASY1 enrichment along *A. thaliana* chromosomes, increasing from the telomeres to the centromeres (Lambing et al., 2020b). Interestingly, *asy1*/+ heterozygotes maintained total CO numbers, but genome-wide mapping revealed that COs were redistributed toward the telomeres at the expense of the pericentromeres (Lambing et al., 2020b). Immunocytology of *asy1*/+ showed that although ASY1 appeared to form a continuous signal along chromosomes and full pairing and synapsis were achieved, ASY1 signal intensity in early prophase I was reduced by 21% compared to wild type (Lambing et al., 2020b). This led to the proposal that *A. thaliana* ASY1 antagonizes telomere-led recombination and promotes spaced CO formation along chromosomes via interference.

This interpretation of ASY1 function in Arabidopsis poses interesting questions regarding the distinctive distal enrichment of ASY1 signal we observed in early prophase I of wheat. It is possible that this feature reflects the more advanced state of axis development in the sub-telomeric regions of chromosomes at this stage, in addition to the chromosome ends being brought together by the formation of a more prominent bouquet in wheat (Martínez-Pérez et al., 1999; Armstrong et al., 2001). Interestingly, ASY1 ChIP-seq analysis of hexaploid wheat (Chinese Spring) revealed a similar pronounced distal enrichment toward the telomeres (Tock et al., submitted). Moreover, the ChIP-seq data also revealed a slight enrichment of ASY1 in the centromeric regions, consistent with our cytological observations of increased ASY1 signal intensity at paired centromeres in pachytene. It is therefore tempting to speculate that ASY1 dosage may influence CO distribution in wheat, as it does in Arabidopsis, although further investigation will be required to establish this. The complex interplay between the meiotic axis proteins, chromatin environment and recombination (Lambing et al., 2020a; Tock et al., submitted) promises to be an interesting area of future wheat research, especially given the contrasting chromatin and recombination landscapes in wheat compared to Arabidopsis.

In summary, this study involved a detailed cytological analysis of meiotic prophase I progression in the hexaploid wheat, Cadenza, providing insights into possible factors influencing the distal bias of COs. We believe it provides a useful framework for future functional studies and initiatives to manipulate recombination in wheat.

## Data Availability Statement

The original contributions presented in the study are included in the article/Supplementary Material, further inquiries can be directed to the corresponding author/s.

## Author Contributions

KO, JH, IH, KE, FCHF, and ESM designed the research. KO and UA performed the experiments. KO, UA, and ESM analyzed the data. KO and FCHF wrote the first draft of the manuscript. All authors contributed to the article and approved the submitted version.

## Conflict of Interest

The authors declare that the research was conducted in the absence of any commercial or financial relationships that could be construed as a potential conflict of interest.
