# Supplementary material for: Distal Bias of Meiotic Crossovers in Hexaploid Bread Wheat Reflects Spatio-Temporal Asymmetry of the Meiotic Program
Source: Front Plant Sci. 2021 Feb 12;12:631323. doi: 10.3389/fpls.2021.631323 (PMC7928317; doi:10.3389/fpls.2021.631323)
Supplement: Supplementary file 1 [file Presentation_1.PPTX]

## Slide 1
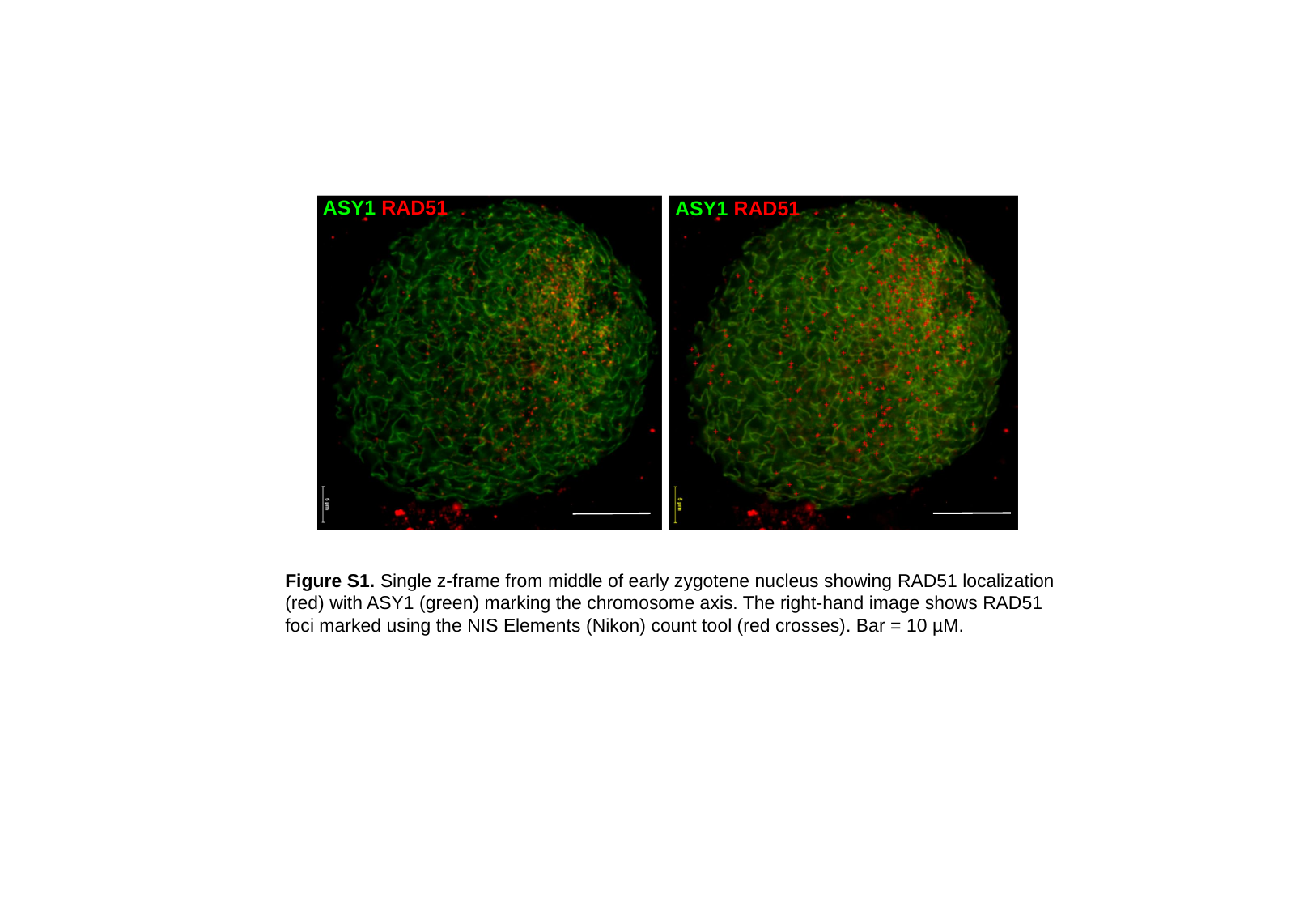

ASY1 RAD51
ASY1 RAD51
Figure S1. Single z-frame from middle of early zygotene nucleus showing RAD51 localization (red) with ASY1 (green) marking the chromosome axis. The right-hand image shows RAD51 foci marked using the NIS Elements (Nikon) count tool (red crosses). Bar = 10 µM.

## Slide 2
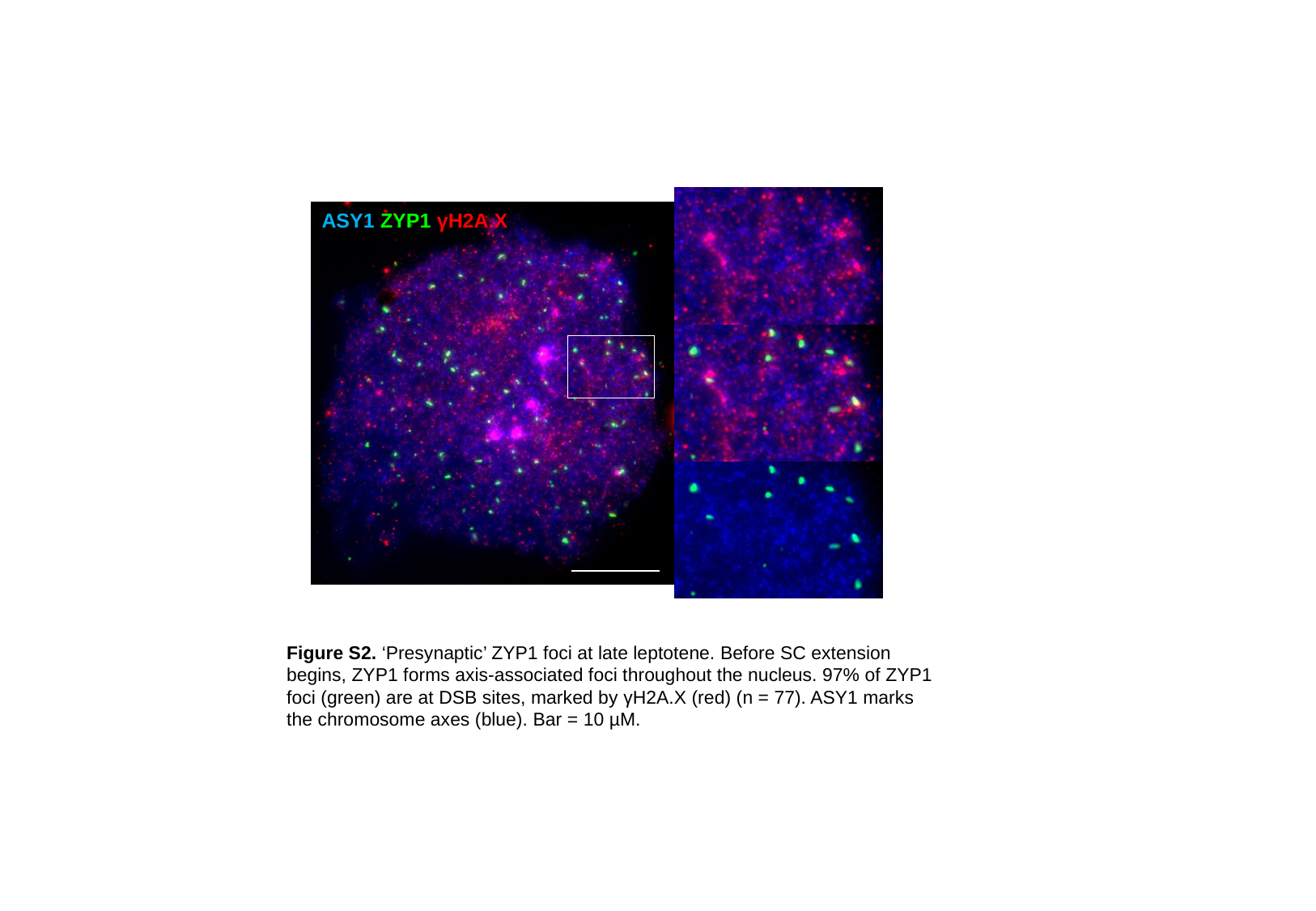

ASY1 ZYP1 γH2A.X
ASY1 ZYP1 γH2A.X
Figure S2. ‘Presynaptic’ ZYP1 foci at late leptotene. Before SC extension begins, ZYP1 forms axis-associated foci throughout the nucleus. 97% of ZYP1 foci (green) are at DSB sites, marked by γH2A.X (red) (n = 77). ASY1 marks the chromosome axes (blue). Bar = 10 µM.

## Slide 3
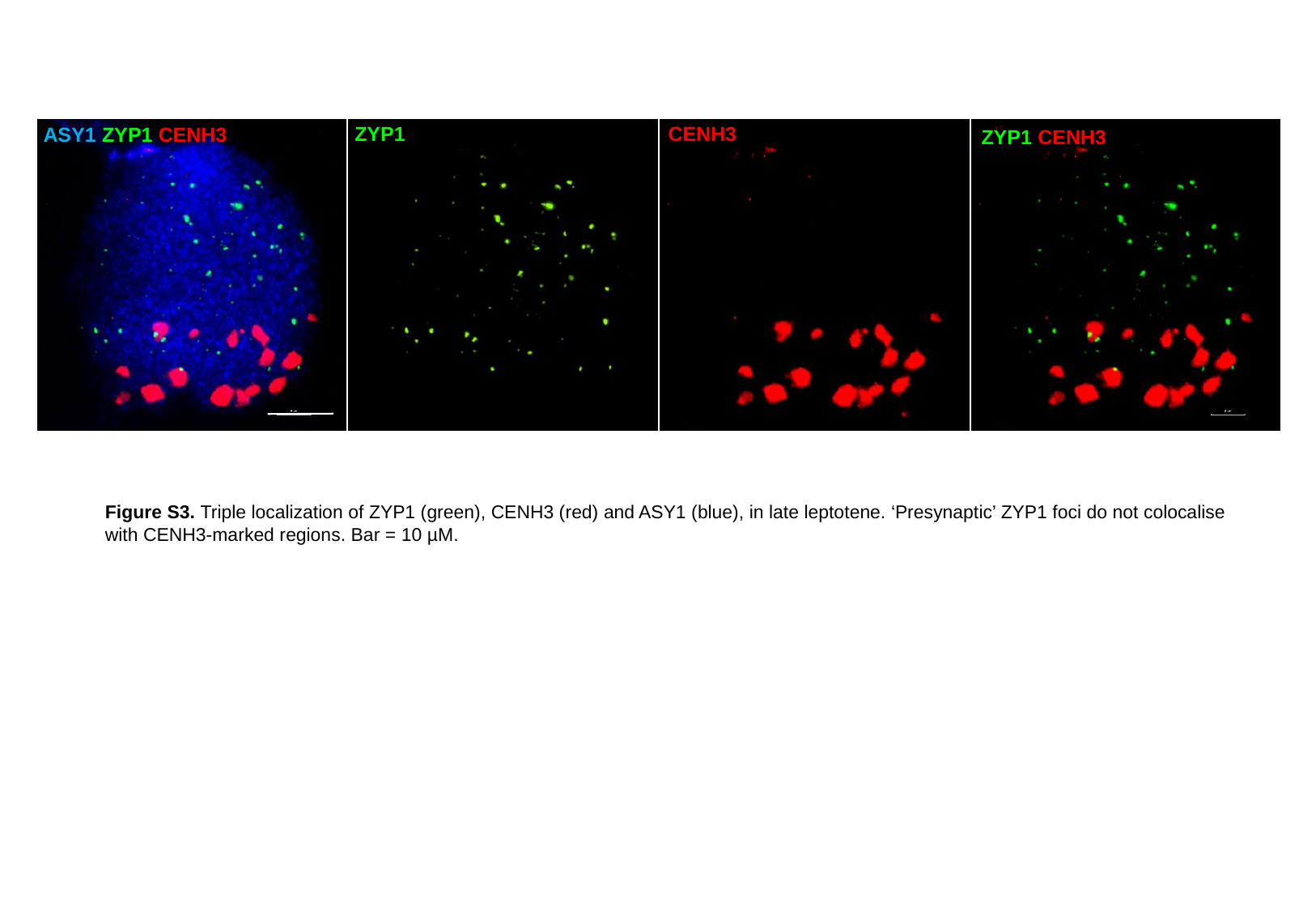

CENH3
ZYP1
ASY1 ZYP1 CENH3
ZYP1 CENH3
Figure S3. Triple localization of ZYP1 (green), CENH3 (red) and ASY1 (blue), in late leptotene. ‘Presynaptic’ ZYP1 foci do not colocalise with CENH3-marked regions. Bar = 10 µM.

## Slide 4
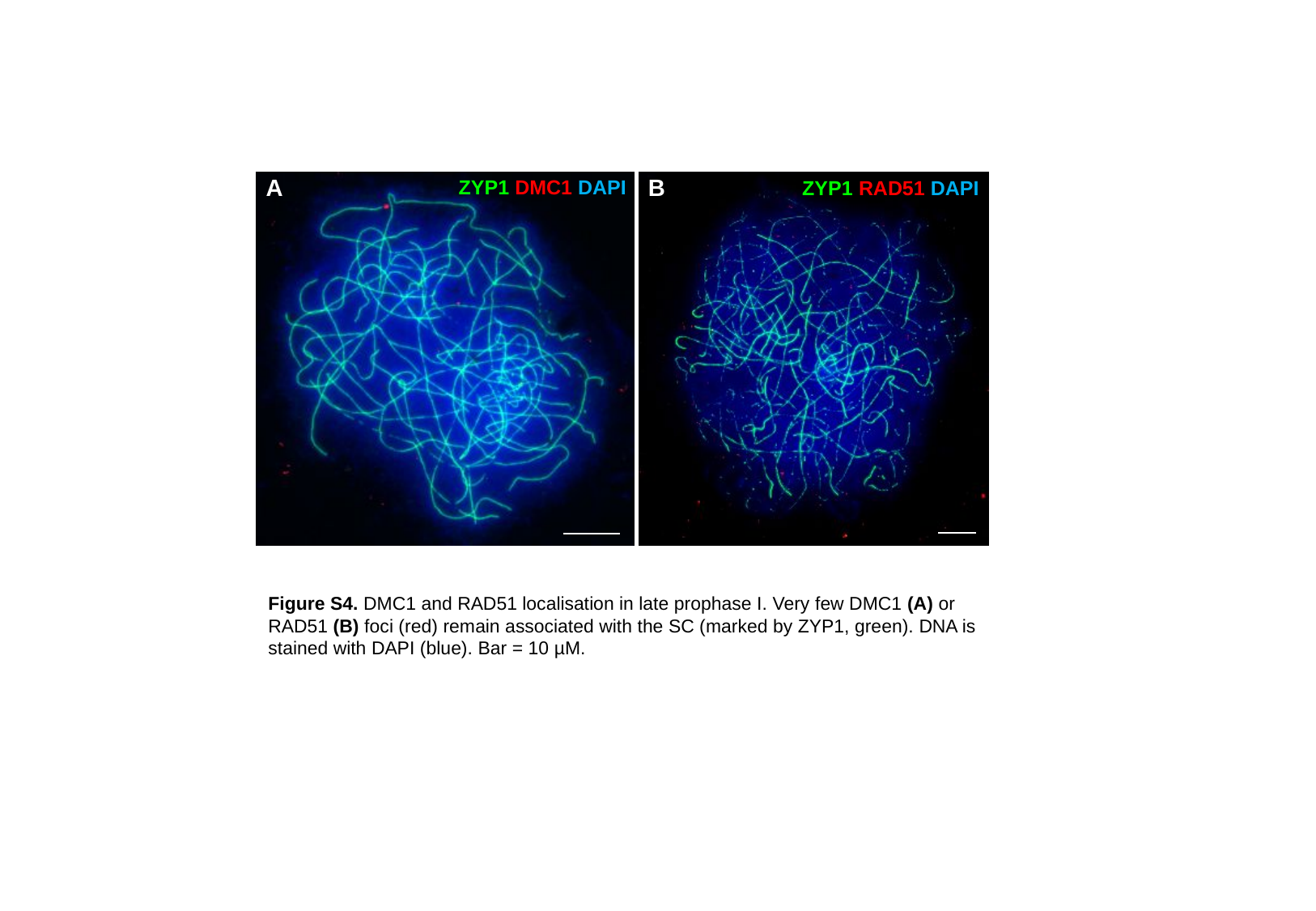

B
A
ZYP1 DMC1 DAPI
ZYP1 RAD51 DAPI
Figure S4. DMC1 and RAD51 localisation in late prophase I. Very few DMC1 (A) or RAD51 (B) foci (red) remain associated with the SC (marked by ZYP1, green). DNA is stained with DAPI (blue). Bar = 10 µM.

## Slide 5
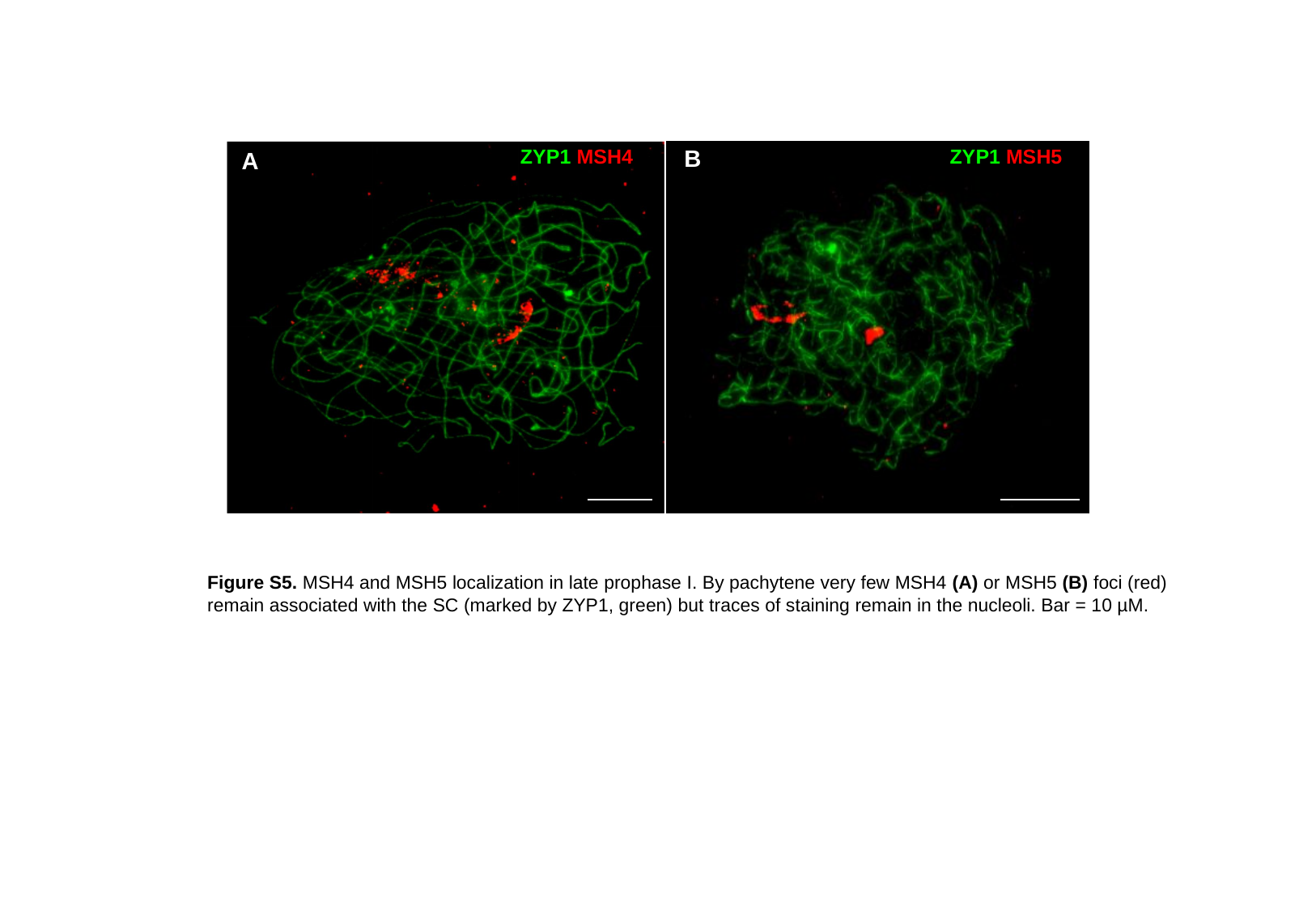

B
ZYP1 MSH5
ZYP1 MSH4
A
Figure S5. MSH4 and MSH5 localization in late prophase I. By pachytene very few MSH4 (A) or MSH5 (B) foci (red) remain associated with the SC (marked by ZYP1, green) but traces of staining remain in the nucleoli. Bar = 10 µM.

## Slide 6
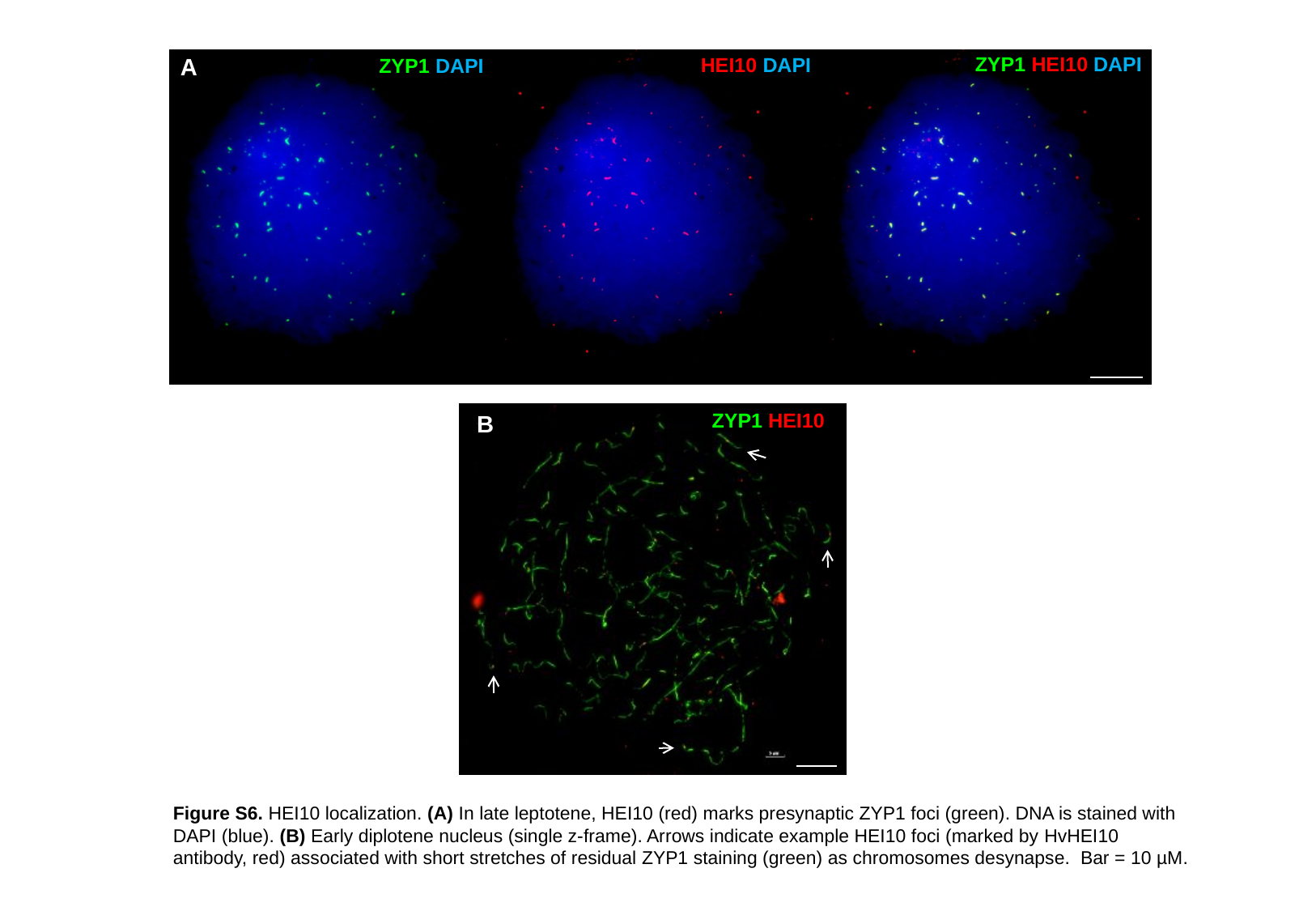

ZYP1 HEI10 DAPI
HEI10 DAPI
A
ZYP1 DAPI
ZYP1 HEI10
B
Figure S6. HEI10 localization. (A) In late leptotene, HEI10 (red) marks presynaptic ZYP1 foci (green). DNA is stained with DAPI (blue). (B) Early diplotene nucleus (single z-frame). Arrows indicate example HEI10 foci (marked by HvHEI10 antibody, red) associated with short stretches of residual ZYP1 staining (green) as chromosomes desynapse. Bar = 10 µM.

## Slide 7
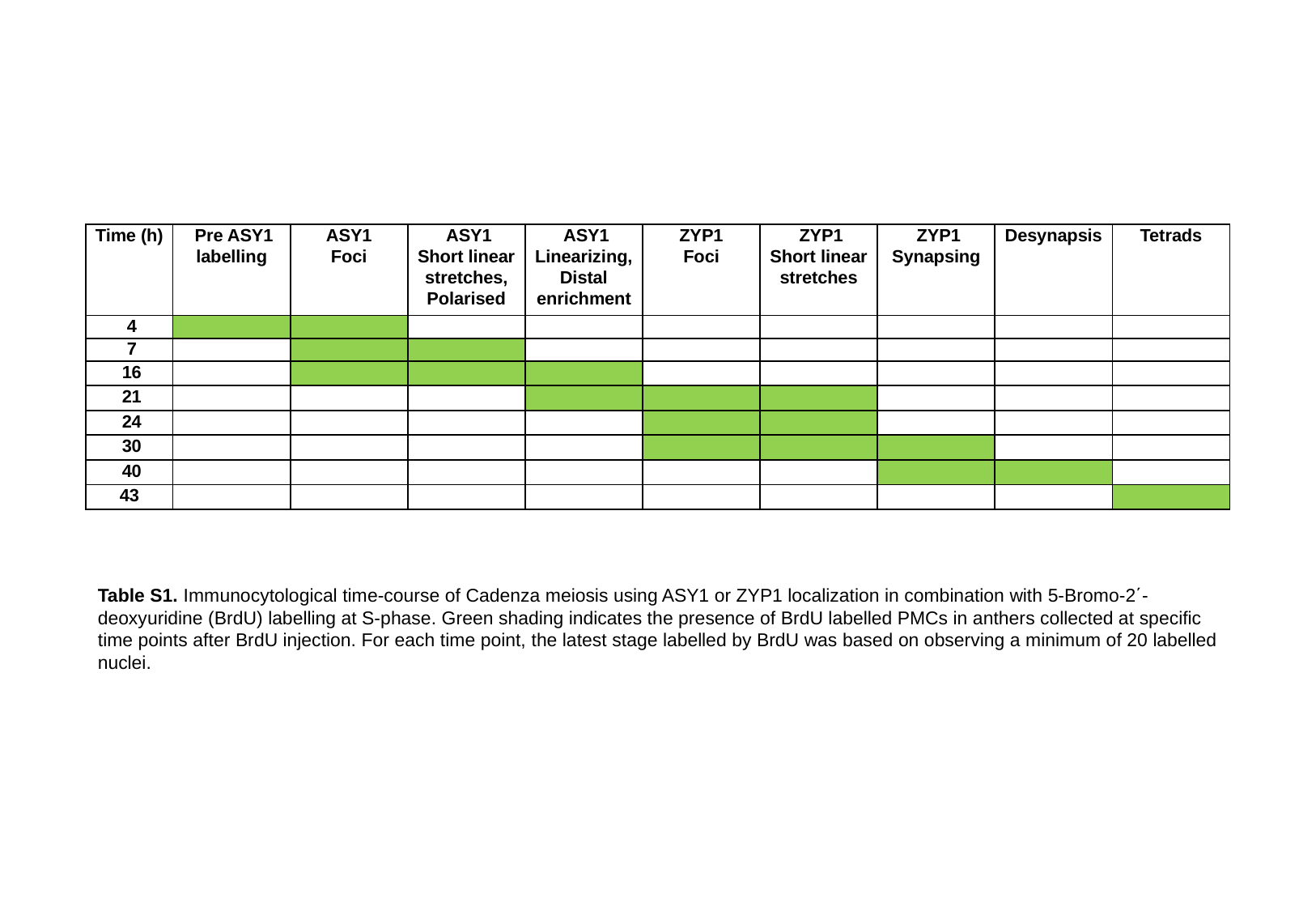

| Time (h) | Pre ASY1 labelling | ASY1 Foci | ASY1 Short linear stretches, Polarised | ASY1 Linearizing, Distal enrichment | ZYP1 Foci | ZYP1 Short linear stretches | ZYP1 Synapsing | Desynapsis | Tetrads |
| --- | --- | --- | --- | --- | --- | --- | --- | --- | --- |
| 4 | | | | | | | | | |
| 7 | | | | | | | | | |
| 16 | | | | | | | | | |
| 21 | | | | | | | | | |
| 24 | | | | | | | | | |
| 30 | | | | | | | | | |
| 40 | | | | | | | | | |
| 43 | | | | | | | | | |
Table S1. Immunocytological time-course of Cadenza meiosis using ASY1 or ZYP1 localization in combination with 5-Bromo-2΄-deoxyuridine (BrdU) labelling at S-phase. Green shading indicates the presence of BrdU labelled PMCs in anthers collected at specific time points after BrdU injection. For each time point, the latest stage labelled by BrdU was based on observing a minimum of 20 labelled nuclei.
